# Morphological Plasticity of the Retina of Viperidae Snakes Is Associated With Ontogenetic Changes in Ecology and Behavior

**DOI:** 10.3389/fnana.2021.770804

**Published:** 2022-01-26

**Authors:** Juliana H. Tashiro, Dora F. Ventura, Einat Hauzman

**Affiliations:** Department of Experimental Psychology, Psychology Institute, University of São Paulo, São Paulo, Brazil

**Keywords:** visual ecology, opsins, visual acuity, stereology, *Crotalus durissus*, *Bothrops jararaca*, retinal topography

## Abstract

Snakes of the Viperidae family have retinas adapted to low light conditions, with high packaging of rod-photoreceptors containing the rhodopsin photopigment (RH1), and three types of cone-photoreceptors, large single and double cones with long-wavelength sensitive opsins (LWS), and small single cones with short-wavelength sensitive opsins (SWS1). In this study, we compared the density and distribution of photoreceptors and ganglion cell layer (GCL) cells in whole-mounted retinas of two viperid snakes, the lancehead *Bothrops jararaca* and the rattlesnake *Crotalus durissus*, and we estimated the upper limits of spatial resolving power based on anatomical data. The ground-dwelling *C. durissus* inhabits savannah-like habitats and actively searches for places to hide before using the sit-and-wait hunting strategy to ambush rodents. *B. jararaca* inhabits forested areas and has ontogenetic changes in ecology and behavior. Adults are terrestrial and use similar hunting strategies to those used by rattlesnakes to prey on rodents. Juveniles are semi-arboreal and use the sit-and-wait strategy and caudal luring to attract ectothermic prey. Our analyses showed that neuronal densities were similar for the two species, but their patterns of distribution were different between and within species. In adults and juveniles of *C. durissus*, cones were distributed in poorly defined visual streaks and rods were concentrated in the dorsal retina, indicating higher sensitivity in the lower visual field. In adults of *B. jararaca*, both cones and rods were distributed in poorly defined visual streaks, while in juveniles, rods were concentrated in the dorsal retina and cones in the ventral retina, enhancing sensitivity in the lower visual field and visual acuity in the upper field. The GCL cells had peak densities in the temporal retina of *C. durissus* and adults of *B. jararaca*, indicating higher acuity in the frontal field. In juveniles of *B. jararaca*, the peak density of GCL cells in the ventral retina indicates better acuity in the upper field. The estimated visual acuity varied from 2.3 to 2.8 cycles per degree. Our results showed interspecific differences and suggest ontogenetic plasticity of the retinal architecture associated with changes in the niche occupied by viperid snakes, and highlight the importance of the retinal topography for visual ecology and behavior of snakes.

## Introduction

The highly diverse group of Snakes, with more than 3,800 species (Uetz et al., 2020) has a fascinating diversity of retinal morphology, especially regarding the photoreceptor types (Walls, 1942; Underwood, 1967a,^1970^; Caprette, 2005; Hauzman et al., 2017; Hauzman, 2020). This group represents a valuable model to test hypotheses of correlation between the types of retinal specialization and species ecology and behavior. The vertebrate retina is formed by layers of cells and nerve plexuses organized in a highly conserved fashion that allows the vertical flow of luminous information from the photoreceptors in the outermost retina toward the ganglion cells in the innermost retina (Ramón y Cajal, 1983). The photoreceptors contain the visual pigments that absorb photons and trigger an enzymatic cascade within the cell. The light energy is converted into neural signals that are transmitted to bipolar cells and from those to the ganglion cells (GCs) that conduct the information to the brain (Ramón y Cajal, 1983).

The density and distribution of cells in the retinas are highly variable among species. Specific regions of higher cell density, the retinal specializations, reflect areas of the visual field that have greater importance for photon uptake, spatial resolution, or other visual functions depending on the cell type (Baden et al., 2020). Two main types of retinal specializations, visual streak and *area centralis*, were described in many vertebrates and are usually associated with the habitat occupied by the species (Hughes, 1977; Moore et al., 2017). Visual streaks are elongated regions of higher cell density that allow a wide screening of the surroundings without the constant need for head and eye movements and are usually associated with the use of open environments. *Areae centrales* are concentric regions of higher cell density usually found in species that occupy closed environments such as forests, where the horizon is obstructed by vegetation (Hughes, 1977; Collin, 2008; Moore et al., 2017).

In Snakes, despite the ecological diversity of the group, a very limited number of studies investigated the organization of neurons in the retinas (Wong, 1989; Hart et al., 2012; Hauzman et al., 2014, 2018). Different types of specializations were described even among sympatric and closely related species. In marine Elapidae snakes the GCs are arranged in horizontal streaks that might enable a better view of the open ocean environment (Hart et al., 2012), and in two out of three marine species analyzed by Hart et al. (2012), an additional *area centralis* in the ventral retina was associated with specific foraging strategies. In the arboreal Dipsadidae snake *Philodryas olfersii*, the photoreceptors and GC are arranged in horizontal streaks, while in the close-related ground-dwelling *Philodryas patagoniensis*, these neurons are concentrated in a ventral *area centralis*, indicating better spatial resolution of the upper visual field (Hauzman et al., 2014). In a comparative study of the distribution of GCs of diurnal and nocturnal Dipsadidae snakes, it was suggested that the type of specialization may also be associated with daily activity patterns and foraging strategies (Hauzman et al., 2018), in which diurnal species that actively forage during the day display visual streaks, while nocturnal species or those that feed on slow-moving prey have *area centralis* in different regions of the retinas (Hauzman et al., 2018). These studies revealed the variability of adaptations of the visual structures of snakes, and indicate that different selective forces may shape the retinal architecture irrespective of phylogenetic blueprints.

The Viperidae family represents a valuable model for investigating adaptations of the visual structures due to the diversity of species and habitats occupied, predation strategies with accurate strike performances (Reiserer, 2002; Chen et al., 2017; Schraft and Clark, 2019), and an elaborate thermosensitive sensory system in pitviper species (subfamily Crotalinae) integrated with inputs from visual neurons in the tectum (Hartline et al., 1978). Additionally, some viperid species have ontogenetic changes in the niche occupied and thus, represent a unique opportunity to explore how morphological adaptations of the retina might be associated with their visual ecology. Viperids are primarily nocturnal or crepuscular, and their retinas have a predominance of rods, highly sensitive photoreceptors adapted to low light (scotopic) conditions, and three types of cones, photoreceptors responsible for daylight (photopic) vision. The rods contain the typical rhodopsin (RH1) photopigment, and cones are classified as single cones and double cones sensitive to medium/long wavelengths, with the LWS photopigment, and single cones sensitive to short wavelengths, with the SWS1 photopigment (Bittencourt et al., 2019; Gower et al., 2019).

**FIGURE 1:**
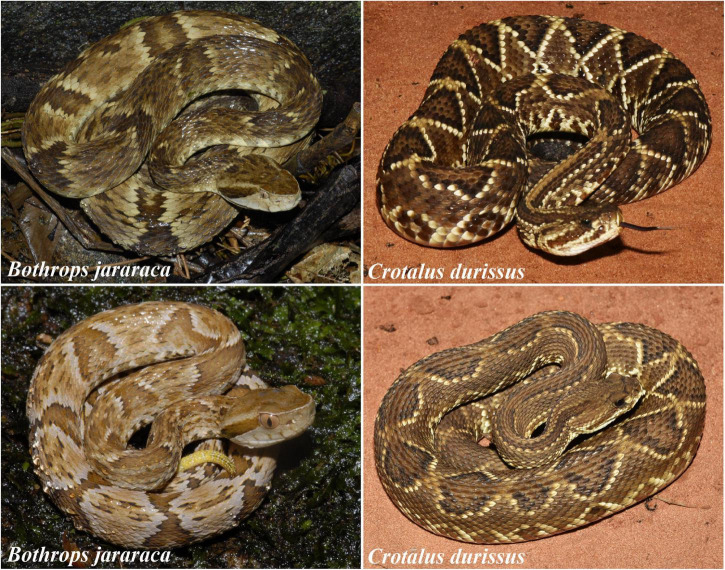
Photographs of adults (upper images) and juveniles (lower images) of *Bothrops jararaca* and *Crotalus durissus*. The whitetail observed in the juvenile of *B. jararaca* (lower left) is used for caudal luring. Photographs: Marcio Roberto Costa Martins.

In this study, we compared the density and distribution of photoreceptors and ganglion cell layer (GCL) cells in the retinas of two pitvipers. The rattlesnake, *Crotalus durissus* (Figure 1), is a terrestrial snake that inhabits open fields of the Cerrado, a Brazilian savannah-like habitat, and actively search for places to hide before using the sit-and-wait hunting strategy to ambush rodents (Salomão et al., 1995; Sawaya et al., 2008; Tozetti and Martins, 2008, 2013). The lancehead, *Bothrops jararaca* (Figure 1), inhabits predominantly forested areas of the Atlantic Rain Forest and has ontogenetic changes in niche occupied and in behavior. Adults are terrestrial and use similar hunting strategies as rattlesnakes to prey on mammals. Juveniles are semi-arboreal and use the sit-and-wait strategy and caudal luring to attract ectothermic vertebrates, mainly anurans (Sazima, 1991, 1992, 2006; Marques and Sazima, 2004). We hypothesized that the differences in behavior and niche occupied by juveniles and adults of *B. jararaca* might be associated with rearrangements of the retinal architecture according to specific visual needs. With a stereological approach to quantify neurons in whole-mounted retinas, we observed differences in the density and distribution of cells between species, especially regarding the proportion and distribution of rods and cones, and differences in the retinal topography of juveniles and adults of *B. jararaca* that might reflect ontogenetic changes in the visual ecology. This study demonstrates that the habitat occupied by snakes and their foraging strategies are associated with different patterns of neuron distribution in the retina, which highlights the importance of their retinal specializations for visually guided behaviors.

## Materials and Methods

### Snakes

Snakes (*n* = 33) were provided by the Butantan Institute, São Paulo, Brazil, and were euthanized with a lethal injection of sodium thiopental (100 mg/kg). Animal procedures were in accordance with ethical principles of animal management and experimentation established by the Brazilian Animal Experiment College (COBEA). The project was approved by the Ethics Committee of Animal Research of the Psychology Institute, University of São Paulo, Brazil. Individuals were classified as adults or juveniles based on the snout-vent length (SVL) and body mass (Supplementary Table 1; Sazima and Abe, 1991; Almeida-Santos, 2005; de Moraes, 2008; Barros, 2011; Fiorillo et al., 2020a).

### Tissue Processing

Following euthanasia, the eyes were enucleated, their axial lengths were measured, and a small radial incision was made in the dorsal region for later orientation. The corneas were removed and the eyecups were fixed in 4% paraformaldehyde (PFA) diluted in phosphate buffer (PB) 0.1 M, for 3 h. The retinas were carefully dissected and maintained in PB 0.1 M at 4°C. When needed, the retinas were bleached in 10% hydrogen peroxide diluted in PB 0.1 M, for 24–48 h, at room temperature, prior to immunohistochemistry or Nissl procedures.

### Immunohistochemistry

For immunohistochemistry, free-floating retinas were preincubated in 10% normal goat serum (Jackson ImmunoResearch, West Grove, United States) or 10% normal donkey serum (Sigma-Aldrich, St. Louis, MO, EUA), diluted in PB 0.1 M with 0.3% Triton X-100, for 1 h, at room temperature. The retinas were incubated with primary antibodies (Table 1) diluted in PB 0.1 M with 0.3% Triton X-100, for 3 days, at 4°C. The following antibodies were used: rabbit anti-SWS1 opsin (Sigma-Aldrich, AB5407; 1:200), rabbit anti-LWS opsin (Sigma-Aldrich, AB5405; 1:200), and for double immunofluorescence labeling, a mixture of the antibodies goat anti-SWS1 opsin (Santa Cruz Biotechnology, sc-14363; 1:200) and rabbit anti-LWS opsin (Sigma-Aldrich; AB5407; 1:200). The retinas were washed in PB 0.1 M with 0.3% Triton X-100 and incubated for 2 h with the secondary antibodies, at room temperature, protected from light: tetramethylrhodamine (TRITC)-conjugated goat anti-rabbit (immunoglobulin G, whole molecules; Jackson Immunoresearch Laboratories; 1:200), and for double-labeled retinas, a combination of TRITC-conjugated donkey anti-goat with Alexa Fluor^®^ 488-conjugated donkey anti-rabbit (immunoglobulin G, whole molecule; Jackson Immunoresearch Laboratories; 1:200). The retinas were rinsed in 0.1 M PB, flat-mounted on glass slides with the photoreceptor layer facing up, mounted with Vectashield (Vector Laboratories Inc. California, United States), and observed under a fluorescent microscope (Leica DM5500B), with a set of filters for TRITC (excitation green, emission red) and Alexa Fluor^®^ 488 (excitation blue, emission green).

**TABLE 1:** Primary and secondary antibodies used to label cones in retinas of snakes.

Antibody	Immunogen	Source, host and catalog no.
**Primary antibody**		
Blue opsin (OPN1SW)	Human (*Homo sapiens*) blue-sensitive opsin NKQFQACIMKMVCG KAMTDESDTCSSQKTEV STVSSTQVGPN	Merck Millipore (Germany). Rabbit polyclonal. Cat#AB5407
Blue opsin (OPN1SW)	Human (*Homo sapiens*) blue-sensitive opsin EFYLFKNISSVGP WDGPQYH	Santa Cruz Biotech. (Germany). Goat polyclonal. Cat#sc14363
Red/Green opsin (OPN1LW)	Human (*Homo sapiens*) red/green–sensitive opsin RQFRNCILQLFGKK VDDGSELSSASKTEV SSVSSVSPA	Merck Millipore (Germany). Rabbit polyclonal. Cat#AB5405
**Secondary antibody**		
Goat anti-rabbit IgG + TRITC	Heavy and light chains of gamma immunoglobulins	Jackson Immunoresearch Lab. (EUA). Goat. Cat#AB2337926
Donkey anti-goat IgG + Alexa Fluor^®^ 488	Heavy and light chains of gamma immunoglobulins	Jackson Immunoresearch Lab. (EUA). Donkey. Cat#AB2340400
Donkey anti-rabbit IgG + TRITC	Heavy and light chains of gamma immunoglobulins	Jackson Immunoresearch Lab. (EUA). Donkey. Cat#AB2340588

### Antibody Characterization and Specificity

**FIGURE 2:**
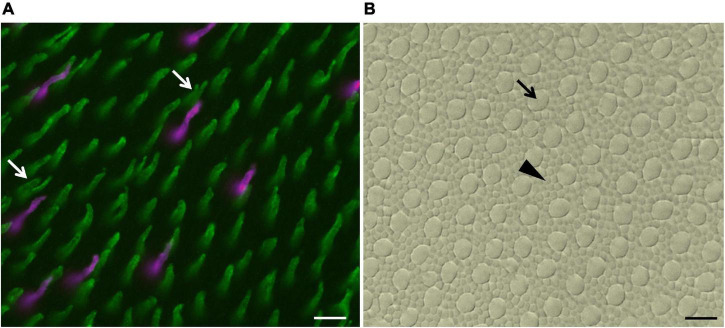
Images of a counting field of a whole-mounted retina of *B. jararaca* double-labeled with anti-SWS1 opsin and anti-LWS opsin antibodies. **(A)** Outer segments of LWS cones (green), including single cones and double cones (white arrows), and of SWS1 cones (magenta). **(B)** Mosaic of photoreceptors of the same field shown in **(A)**, viewed under bright light: inner segments of rods (black arrowhead) and cones (black arrow). Scale bars 10 μm.

Immunohistochemistry procedures were performed with polyclonal antibodies raised in rabbits against the last 42 amino acids of the C-terminal of human blue opsin (Sigma-Aldrich; AB5407), against the last 38 amino acids of the C-terminal of human red/green opsins (Sigma-Aldrich; AB5405), or raised in goats against a synthetic peptide with 20 amino acids of human blue opsin (Santa Cruz Biotechnology; sc-14363) (Table 1). The specificity of the antibodies for snakes was described previously (Hauzman et al., 2014, 2017; Bittencourt et al., 2019; Gower et al., 2019). Double labeling with the antibodies against SWS1 and against LWS opsins showed differential labeling of distinct photoreceptor populations (Figure 2), further indicating the specificity of both antibodies for particular types of cones. We also assessed the specificity of the two anti-SWS1 antibodies, by incubating 12 μm retinal sections of *B. jararaca* and *C. durissus* obtained at −25°C with a cryostat (Leica, CM1100; Nussloch, Germany), with a mixture of both antibodies, rabbit anti-SWS1 (AB5407; 1:200) and goat anti-SWS1 (sc14363; 1:200). Immunofluorescence visualization showed a small number of small single cones labeled by both antibodies (data not shown), indicating the specificity of both antibodies against SWS1 cones.

### Nissl Staining

We used Nissl staining technique in whole-mounted retinas to analyze the population of GCL cells. Small radial incisions were made in the dissected retinas to allow them to be flat-mounted onto gelatinized glass slides, with the GCL facing up. The retinas were incubated with paraformaldehyde vapors overnight, at room temperature, for enhancing the adherence to the slide and to increase the differentiation of ganglion cells during staining (Coimbra et al., 2006). The tissues were rehydrated in decreasing ethanol series (95, 70, 50%), rinsed in distilled water acidified with glacial acetic acid, stained in aqueous solution of 2% cresyl violet for approximately 1 min, dehydrated in increasing concentrations of ethanol, cleared in xylene, and coverslipped with DPX (Sigma-Aldrich. St. Louis, MO, EUA).

**FIGURE 3:**
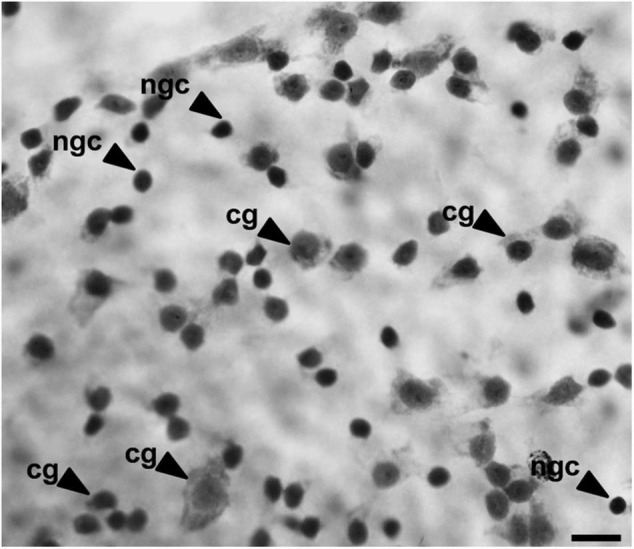
Image of the GCL cells of *B. jararaca* indicating the morphological criteria used to distinguish ganglion cells (gc), with polygonal cell bodies, from other cell populations (ngc, non-ganglion cell), with a well-defined circular shape and dense nucleus. Scale bar: 10 μm.

To analyze the density and distribution of GCL cells, we used the cytological criteria proposed by Wong (1989) to distinguish ganglion cells from amacrine and glial cells. The ganglion cells were differentiated by their larger polygonal soma, abundant Nissl substance in the cytoplasm, and a prominent nucleolus (Figure 3; Hart et al., 2012). Glial cells were differentiated by smaller soma size and a round and darkly stained profile, and amacrine cells were identified by a smaller and circular profile with a more darkly stained nucleus compared to ganglion cells (Hart et al., 2012). The glial and amacrine cells were not counted.

### Stereological Assessment of the Density and Distribution of Retinal Neurons

The density and distribution of retinal neurons were accessed using a stereological approach based on the optical fractionator method (West et al., 1991), modified for retinal whole-mounts (Coimbra et al., 2009, 2012), using a motorized microscope (DM5500B, Leica Microsystems, Germany), connected to a computer running the Stereo Investigator software (MicroBrightField, Colchester, VT). The coordinates of the outer edges of the retinas were obtained using a 5x/NA 0.15 objective. Approximately 200 counting frames were positioned in a random fashion covering the entire area of the retina. Cells were counted when laying entirely within the counting frame or when intersected the acceptance lines, without touching the rejection lines (Gundersen, 1997). The area of the counting frame and the sampling grids varied according to the cell types and the retinal area, and were defined in pilot experiments, in order to obtain an acceptable Scheaffer coefficient of error (CE) (<0.10) (Scheaffer et al., 1996). The stereological parameters used to estimate the number of photoreceptors and GCL cells of each retina are described in Supplementary Tables 2, 3. To estimate the total population of neurons (N_total_), we considered the area of sampling fraction (asf) according to the algorithm: N_total_ = ∑*Q*×1/*asf*, where ∑*Q* is the sum of the total number of neurons counted and the area of sampling fraction is the ratio between the counting frame and the sampling grid (Coimbra et al., 2009).

### Anatomical Estimates of the Visual Acuity

**FIGURE 4:**
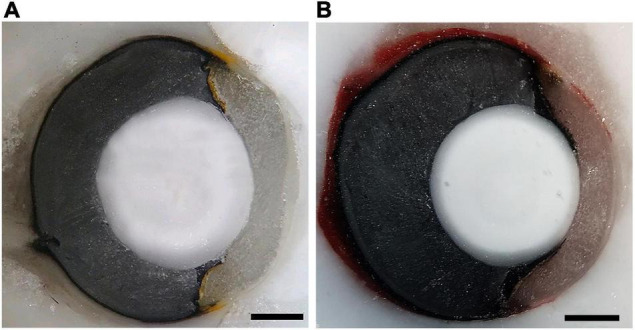
Cryosectioned eye from adults of **(A)** *B. jararaca* and **(B)** *C. durissus*. Scale bars: 1 mm.

We estimated the upper limits of spatial resolving power based on the peak density of GCL cells and the presumed focal distance of the eyes. The focal lengths were estimated by freezing and sectioning eyes from one adult and one juvenile individual of each species. The fresh eyes were enucleated and rapidly frozen, embedded in Tissue-Tek OCT compound (Sakura Finetechnical Co., Tokyo, Japan), and the blocks were sectioned at −25°C on a cryostat (Leica CM1100; Nussloch, Germany). Photographs of the blocks were taken every 12 μm, with a camera (Axio CamMR, Carl ZeissVision, Germany) coupled to a stereomicroscope (SMZ775-T, NIKON, Japan), and a computed running the Axio Vision 4.1 software (Carl Zeiss, Germany). The areas of the lens and eyes were measured using the ImageJ software (NIH, Bethesda, United States) and the photographs that had the larger lens diameters were identified and used for optical measurements. Measurements of the axial length of the eye, lens axial diameter, and posterior nodal distance (PND) that represents the focal length and corresponds to the distance from the center of the lens to the retina-choroid border were taken along the optical axis, which was located by connecting the geometric centers of the optical components (Lisney and Collin, 2008). In *B. jararaca*, the focal length corresponded to 50% of the adult eye axial length of the adult (Figure 4A) and 67% of the juvenile. In *C. durissus* the focal distance corresponded to 60% of eyes axial length of both adult (Figure 4B) and juvenile. Those values were used to estimate the focal distance of the eyes used for GCL cell counts.

We estimated the upper limits of spatial resolution considering two possible arrangements of the ganglion cells, a hexagonal and a square array. The distance *d* subtended as 1 degree on the retina was determined from the PND and the equation *d* = (2π*PND*)/360 (Pettigrew et al., 1998). Considering a hexagonal array, we estimated the average spacing between cells (S) using the formula $S^{2}=2/\left(D\sqrt{3}\right)$, where D is the peak density of GCL cells in cells/mm^2^. The maximum spatial frequency (v) (Nyquist) of a sinusoidal grating (Snyder and Miller, 1977) that has resolution with this cellular arrangement was calculated as $v=1/S\sqrt{3}$. This value was multiplied by the distance *d*, to obtain the spatial resolution in cycles per degree (cpd). In a second approach, considering that the ganglion cells might be organized in a square distribution, we estimated the linear density of GCL cells (cells/mm^2^) from the square root of the peak density (D), and divided the linear cell density by 2 (because at least 2 cells are required to detect 1 cycle of a given spatial frequency). The resulting value was multiplied by the distance *d* to obtain the visual acuity in cpd (Pettigrew et al., 1998; Coimbra et al., 2013).

To infer the behavioral significance of the estimated visual acuity in an ecological context, we predicted the minimum size of objects that the viperid snakes can spatially resolve (Coimbra et al., 2017). To do so, we estimated the angular distance in the retina that corresponds to one cycle by calculating the inverse of the spatial resolving power (cycles/degree). This value was divided by 2 to obtain the minimum angle of resolution (MAR), which represents the angular distance of the smallest resolvable detail on the retina. Subsequently, using the trigonometric relationship between the MAR and a presumed distance (D), relevant, for instance, for foraging a prey or for predator detection, we estimated the minimum object size (obj), according to the equation: D = *obj*/*tanMar*. According to the Nyquist sampling theorem, because an object needs to be twice the threshold to be spatially resolved, we multiplied the minimum object size by 2 (Marshall, 2000).

### Statistical Analysis

Statistical analyzes were performed with R (version 4.0.2)^1^ and the RStudio software (1.3.959), to compare the total population and mean densities of retinal neurons among the four sampled groups, adults and juveniles of *B. jararaca* and of *C. durissus*, as well as the retinal area, eyes axial length and visual acuity. The normality of the distribution of values in each group was checked using the Shapiro-Wilk test, and homogeneities among groups were analyzed using the Levene test. The non-parametric test of Mann-Whitney for independent samples was applied for comparisons, even when values had normal distribution, due to the low sampling size in each group. Differences were considered significant when *p* < 0.05.

## Results

We analyzed the density and distribution of photoreceptors and GCL cells in 18 retinas of *B. jararaca* (adults: *n* = 8; juveniles: *n* = 10) and 17 retinas of *C. durissus* (adults: *n* = 9; juveniles: *n* = 8) (Tables 2–4). The different populations of cones were identified by immunohistochemistry labeling with antibodies against SWS1 and LWS opsins (Figure 2A). The total photoreceptor population was viewed under bright light and by adjusting the focus of the microscope into the photoreceptor’s inner segments level (Figure 2B). Cones and rods were differentiated by the larger diameters of the inner segments of cones compared to the small and highly packed inner segments of rods (Gower et al., 2019; Figure 2B). For cell counting in each sampling field, the photoreceptors labeled by the antibodies were counted first, then all photoreceptors (cones and rods) were counted under bright light to analyze the proportion of each photoreceptor type (rods, LWS, and SWS1 cones). Single and double LWS cones were not always easily differentiated from each other and were quantified together (Figure 2A).

**TABLE 2:** Stereological assessment of total photoreceptors, rods and cones in retinas of adults and juveniles of *B. jararaca* and *C. durissus*.

Species		Total photoreceptors					Rods						Cones					
	Retinal area (mm^2^)	Estimated population	CE	Mean density cells/mm^2^	Max. density cells/mm^2^	Min. density cells/mm^2^	Estimated population	CE	Mean density cells/mm^2^	Max. density cells/mm^2^	Min. density cells/mm^2^	% of rods	Estimated population	CE	Mean density cells/mm^2^	Max. density cells/mm^2^	Min. density cells/mm^2^	% of cones
***B. jararaca***																		
Bj-A#3-RE	46.7	3,636,618	0.03	77,872	114,400	12,400	3,371,110	0.03	72,187	105,600	9,600	92.7	264,878	0.02	5,672	9,200	400	7.3
Bj-A#4-LE	31.2	2,869,378	0.04	91,967	148,800	12,400	2,709,182	0.04	86,833	141,200	10,400	94.4	158,654	0.04	5,085	10,800	400	5.5
Bj-A#5-RE	53.2	3,807,837	0.03	71,576	109,600	18,400	3,546,488	0.03	66,663	104,400	16,400	93.1	260,680	0.02	4,900	8,000	800	6.8
Bj-A#6-LE	54.0	2,851,821	0.04	52,909	89,200	11,200	2,817,263	0.01	52,268	84,400	6,800	98.8	206,879	0.02	3,838	5,600	400	7.3
Mean ± sd	46.3 ± 10.6	3,291,413 ± 502,399	0.04 ± 0.01	73,581 ± 16,205	115,500 ± 24,743	13,600 ± 3,250	3,111,011 ± 410,304	0.03 ± 0.01	69,488 ± 14,290	108,900 ± 23,627	10,800 ± 4,040	94.8 ± 2.8	264,878 ± 50,245	0.03 ± 0.01	4,874 ± 765	8,400 ± 2,191	500 ± 200	6.7 ± 0.8
Bj-J#2-RE	21.2	3,584,610	0.03	169,085	220,800	32,800	3,354,150	0.03	158,215	210,000	26,000	93.6	230,245	0.01	10,864	15,200	2,800	6.4
Bj-J#3-RE	23.8	3,055,351	0.03	128,376	176,400	48,400	2,842,807	0.03	119,446	167,600	42,800	93.0	212,322	0.02	8,921	12,800	2,400	6.9
Bj-J#4-LE	24.4	2,796,266	0.04	114,601	196,800	26,400	2,633,346	0.04	107,924	186,000	23,200	94.2	257,634	0.01	10,559	16,400	2,000	9.2
Mean ± sd	23.1 ± 1.7	3,145,409 ± 401,814	0.03 ± 0.01	137,354 ± 28,330	198,000 ± 22,224	35,867 ± 11,316	2,943,434 ± 370,788	0.03 ± 0.01	128,528 ± 26,347	187,867 ± 21,262	30,667 ± 10,601	93.6 ± 0.6	233,400 ± 22,820	0.01 ± 0.00	10,113 ± 1,044	14,800 ± 1,833	2,400 ± 400	7.5 ± 1.5
***C. durissus***																		
Cd-A#1-LE	55.1	3,585,315	0.03	65,069	113,600	21,200	3,249,205	0.03	58,969	103,600	18,000	90.6	335,662	0.02	6,092	11,600	1,200	9.4
Cd-A#2-RE	49.7	3,364,254	0.04	67,691	102,000	20,400	3,005,135	0.04	60,465	93,200	18,000	89.3	359,130	0.02	7,226	11,200	1,600	10.7
Cd-A#3-LE	48.3	3,648,600	0.04	75,540	123,600	14,800	3,296,354	0.04	68,248	112,800	12,800	90,3	369,688	0.04	7,654	12,400	1,200	10.1
Cd-A#4-LE	41.8	3,637,157	0.03	87,013	124,000	23,600	3,267,345	0.03	78,166	112,400	20,800	89.8	369,717	0.02	8,845	13,600	1,200	10.2
Cd-A#5-RE	39.5	3,402,368	0.03	87,128	131,200	29,600	3,095,653	0.03	78,371	121,200	23,200	91.0	343,716	0.02	8,702	13,600	1,600	10.1
Mean ± sd	46.9 ± 6.3	3,527,539 ± 134,480	0.03 ± 0.01	76,488 ± 10,400	118,880 ± 11,326	21,920 ± 5,370	3,182,738 ± 126,107	0.03 ± 0.01	68,844 ± 9,297	108,640 ± 10,642	18,560 ± 3,884	90.2 ± 0.7	355,582 ± 15,403	0.02 ± 0.01	7,704 ± 1,132	12,480 ± 1,110	1,360 ± 219	10.1 ± 0.5
Cd-J#1-RE	23.3	3,432,051	0.03	147,298	197,600	32,000	3,085,128	0.03	132,409	190,000	29,600	89.9	351,105	0.03	15,069	23,200	2,400	10.2
Cd-J#5-RE	22.4	2,761,716	0.03	125,533	220,000	41,600	2,503,403	0.04	111,759	198,000	13,200	90.6	312,409	0.01	13,947	22,800	2,000	11.3
Cd-J#6-LE	25.0	3,084,595	0.03	123,384	188,800	26,800	2,751,033	0.03	110,041	173,200	22,000	89.2	344,946	0.01	13,798	19,600	1,200	11.2
Mean ± sd	23.6 ± 1.3	3,092,787 ± 335,242	0.03 ± 0.00	132,071 ± 13,230	202,133 ± 16,086	33,467 ± 7,508	2,779,855 ± 291,932	0.03 ± 0.00	118,070 ± 12,448	187,067 ± 12,658	21,600 ± 8,207	89.9 ± 0.7	336,153 ± 20,793	0.02 ± 0.01	14,471 ± 695	21,867 ± 1,973	1,867 ± 611	10.9 ± 0.6

*CE, Scheaffer’s coefficient of error; sd, standard deviation; RE, right eye; LE, left eye.*

### Population of Photoreceptors

**FIGURE 5:**
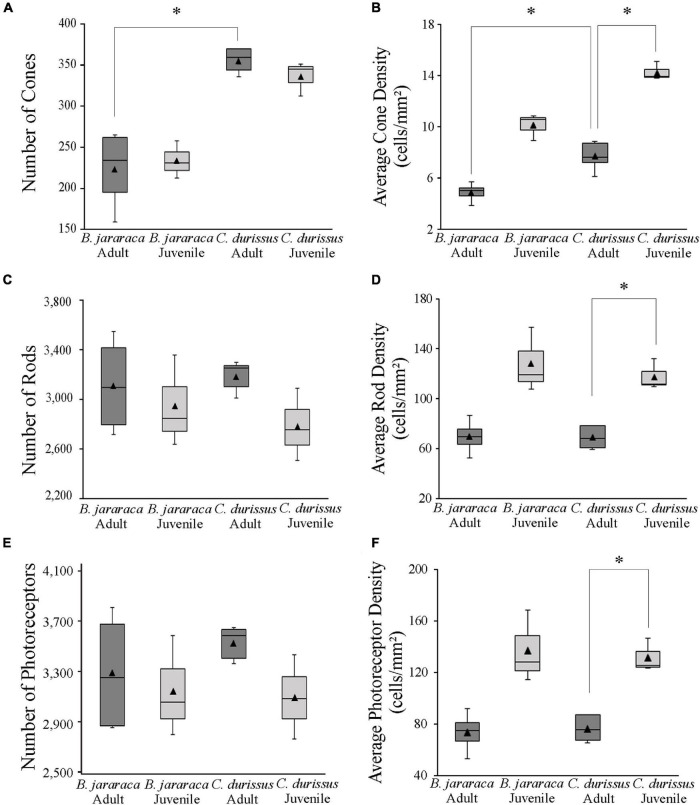
Boxplot representations of the medians (thick black lines) and quartiles (boxes) of the total number and mean density of **(A,B)** cones, **(C,D)** rods, and **(E,F)** photoreceptors in retinas of adults and juveniles of *B. jararaca* and *C. durissus*. Mean density values are represented by the triangles. The values should be multiplied by 10^3^. Groups with statistically significant differences are indicated by asterisk (*p < 0.05).

The total population of photoreceptors was estimated from the whole-mounted retinas of *B. jararaca* (*n* = 7) and *C. durissus* (*n* = 8). Adults and juveniles of both species had similar average total photoreceptors values, ranging from 3,092,787 ± 335,242 (median: 3,084,595) to 3,527,539 ± 134,480 (median: 3,585,315) cells in juveniles and adults of *C. durissus*, respectively (Figure 5 and Table 2). The mean density of photoreceptors, however, was higher in juveniles of both species (137,354 ± 28,330 cells/mm^2^; median: 128,376 cells/mm^2^ in *B. jararaca* and 132,071 ± 13,230 cells/mm^2^; median: 125,533 cells/mm^2^ in *C. durissus*) compared to adults (73,581 ± 16,205 cells/mm^2^; median: 74,724 cells/mm^2^ in *B. jararaca* and 76,488 ± 10,400 cells/mm^2^; median: 75,540 cells/mm^2^ in *C. durissus*) (Figure 5 and Table 2).

Rods were predominant and accounted for approximately 90% of the photoreceptors in retinas of both species (Table 2). The total population of rods was similar between adults and juveniles, varying from 2,779,855 ± 291,932 (median: 2,751,033) cells in juveniles of *C. durissus* and 3,182,738 ± 126,107 (median: 3,249,205) cells in adults (Figure 5 and Table 2). The average density of rods was higher in juveniles (128,528 ± 26,347 cells/mm^2^; median: 119,446 cells/mm^2^ in *B. jararaca* and 118,070 ± 12,448 cells/mm^2^; median: 111,759 cells/mm^2^ in *C. durissus*) compared to adults (69,488 ± 14,290 cells/mm^2^; median: 69,425 cells/mm^2^ in *B. jararaca* and 68,844 ± 9,297 cells/mm^2^; median: 68,248 cells/mm^2^ in *C. durissus*) (Figure 5 and Table 2).

The total population of cones represented about 7% of the photoreceptors in *B. jararaca*, and about 10% in *C. durissus* (Table 2), and was similar between adults and juveniles, varying from 233,400 ± 22,82 (median: 230,245) cells in juveniles of *B. jararaca* and 355,582 ± 15,403 (median: 359,130) cells in adults of *C. durissus* (Figure 5 and Table 2). The average density of cones was higher in juveniles (10,113 ± 1,044 cells/mm^2^; median: 10,559 cells/mm^2^ in *B. jararaca* and 14,471 ± 695 cells/mm^2^; median: 13,947 cells/mm^2^ in *C. durissus*) compared to adults (4,874 ± 765 cells/mm^2^; median: 4,993 cells/mm^2^ in *B. jararaca* and 7,704 ± 1,132 cells/mm^2^; median: 7,654 cells/mm^2^ in *C. durissus*) (Figure 5 and Table 2). The mean density of cones was significantly higher in adults of *C. durissus* compared with adults of *B. jararaca* (Figure 5).

**FIGURE 6:**
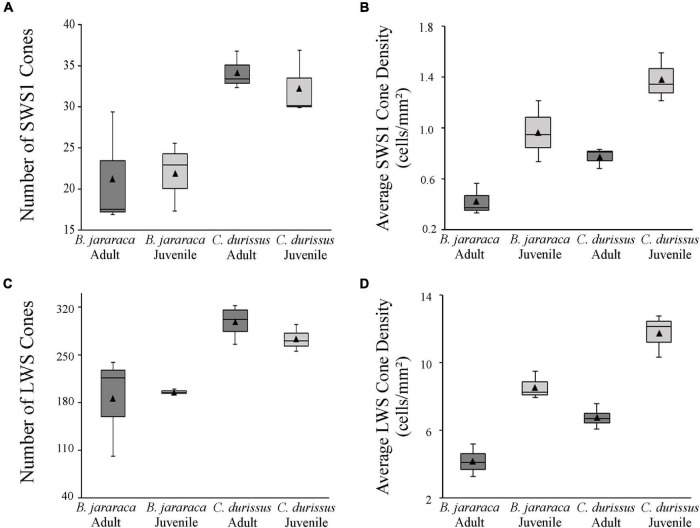
Boxplot representations of the medians (thick black lines) and quartiles (boxes) of the total number and mean density of **(A,B)** small single SWS1 cones and **(C,D)** large single and double LWS cones, in retinas of adults and juveniles of *B. jararaca* and *C. durissus*. The mean density values are represented by the triangles. The values should be multiplied by 10^3^.

The population of SWS1 cones accounted for approximately 10% of the cones in retinas of both species (Table 3). The total population of SWS1 cones was similar between adults and juveniles, and lower in *B. jararaca* (21,655 ± 0,466 cells; adults median: 17,529 cells; juveniles median: 23,007 cells) compared with *C. durissus* (33,354 ± 1,366 cells; adults median: 33,563 cells; juveniles median: 30,126 cells) (Figure 6 and Table 3). The average density was higher in juveniles (960 ± 242 cells/mm^2^; median: 943 cells/mm^2^ in *B. jararaca* and 1,378 ± 195 cells/mm^2^; median: 1,340 cells/mm^2^ in *C. durissus*) compared with adults (414 ± 123 cells/mm^2^; median: 364 cells/mm^2^ in *B. jararaca* and 765 ± 83 cells/mm^2^; median: 803 cells/mm^2^ in *C. durissus*) (Figure 6 and Table 3).

**TABLE 3:** Stereological assessment of SWS1 and LWS cones in retinas of adults and juveniles of *B. jararaca* and *C. durissus*.

Species	SWS1 cones							LWS cones					
	Retinal area (mm^2^)	Estimated population	CE	Mean density cells/mm^2^	Max. density cells/mm^2^	Min. density cells/mm^2^	% of SWS1 cones	Estimated population	CE	Mean density cells/mm^2^	Max. density cells/mm^2^	Min. density cells/mm^2^	% of LWS cones
***B. jararaca***													
Bj-A#3-RE	46.7	16,977	0.03	364	833	31	6.4	238,939	0.03	5,116	8,549	154	90.2
Bj-A#4-LE	31.2	−	−	−	−	−		100,325	0.04	3,216	6,844	133	63.2
Bj-A#5-RE	53.2	29,468	0.03	554	1,142	62	11.3	216,110	0.03	4,062	6,358	370	82.9
Bj-A#6-LE	54.0	17,529	0.04	325	741	31	8.5	−	−	−	−	−	
Mean ± sd	46.3 ± 10.6	21,325 ± 7,058	0.04 ± 0.01	414 ± 123	905 ± 210	41 ± 18	8.7 ± 2.5	185,125 ± 74,321	0.03 ± 0.01	4,131 ± 952	7,250 ± 1,151	219 ± 131	78.8 ± 14.0
Bj-J#2-RE	21.2	25,644	0.04	1,210	2,400	300	11.1	199,050	0.03	9,389	12,900	1,100	86.5
Bj-J#3-RE	23.8	17,303	0.04	727	1,500	200	8.1	194,195	0.03	8,159	11,700	2,200	91.5
Bj-J#4-LE	24.4	23,007	0.03	943	2,300	100	8.9	191,516	0.03	7,849	13,200	2,400	74.3
Mean ± sd	23.1 ± 1.7	21,985 ± 4,263	0.03 ± 0.00	960 ± 242	2.067 ± 493	200 ± 100	9.4 ± 1.5	194,920 ± 3,819	0.03 ± 0.00	8,466 ± 814	12,600 ± 794	1,900 ± 700	84.1 ± 8.8
***C. durissus***													
Cd-A#1-LE	55.1	36,898	0.03	670	1,296	216	11.0	−	−	−	−	−	−
Cd-A#2-RE	49.7	−	−	−	−	−	−	322,641	0.03	6,492	10,370	1,574	89.9
Cd-A#3-LE	48.3	−	−	−	−	−	−	290,315	0.04	6,011	9,599	1,080	78.5
Cd-A#4-LE	41.8	33,563	0.03	803	1,451	247	9.1	313,665	0.03	7,504	11,512	1,636	84.8
Cd-A#5-RE	39.5	32,502	0.03	823	1,605	93	9.5	265,604	0.03	6,724	10,247	1,142	77.4
Mean ± sd	46.9 ± 6.3	34,321 ± 2,294	0.03 ± 0.00	765 ± 83	1,451 ± 155	185 ± 81	9.8 ± 1.0	298,056 ± 25,568	0.04 ± 0.01	6,683 ± 623	10,432 ± 795	1,358 ± 287	82.6 ± 5.8
Cd-J#1-RE	23.3	37,023	0.03	1,589	3,800	200	10.5	294,591	0.04	12,643	19,700	2,200	83.9
Cd-J#5-RE	22.4	30,016	0.05	1,340	2,900	500	9.6	269,625	0.03	12,037	19,400	1,600	86.3
Cd-J#6-LE	25.0	30,126	0.04	1,205	2,600	100	8.7	255,124	0.04	10,205	17,100	1,100	74.0
Mean ± sd	23.5 ± 1.3	32,388 ± 44,014	0.04 ± 0.01	1,378 ± 195	3,100 ± 624	267 ± 208	9.6 ± 0.9	273,113 ± 19,964	0.04 ± 0.00	11,628 ± 1,270	18,733 ± 1,422	1,567 ± 451	81.4 ± 6.5

*CE, Scheaffer’s coefficient of error; sd, standard deviation; RE, right eye; LE, left eye.*

The LWS cones accounted for approximately 85% of the cones (Table 3). The total population of LWS cones was similar between adults and juveniles of both species, and lower in *B. jararaca* (190,022 ± 6,927 cells; adults median: 216,110 cells; juveniles median: 194,195 cells) compared with *C. durissus* (292,585 ± 17,63 cells; adults median: 301,990 cells; juveniles median: 269,625 cells) (Figure 6 and Table 3). In both species the mean density of LWS cones was higher in juveniles (8,466 ± 814 cells/mm^2^; median: 8,159 cells/mm^2^ in *B. jararaca* and 11,628 ± 1,207 cells/mm^2^; median: 12,037 cells/mm^2^ in *C. durissus*) compared with adults (4,131 ± 952 cells/mm^2^; median: 4,062 cells/mm^2^ in *B. jararaca* and 6,683 ± 623 cells/mm^2^; median: 6,608 cells/mm^2^ in *C. durissus*) (Figure 6 and Table 3).

### Photoreceptors Topography

**FIGURE 7:**
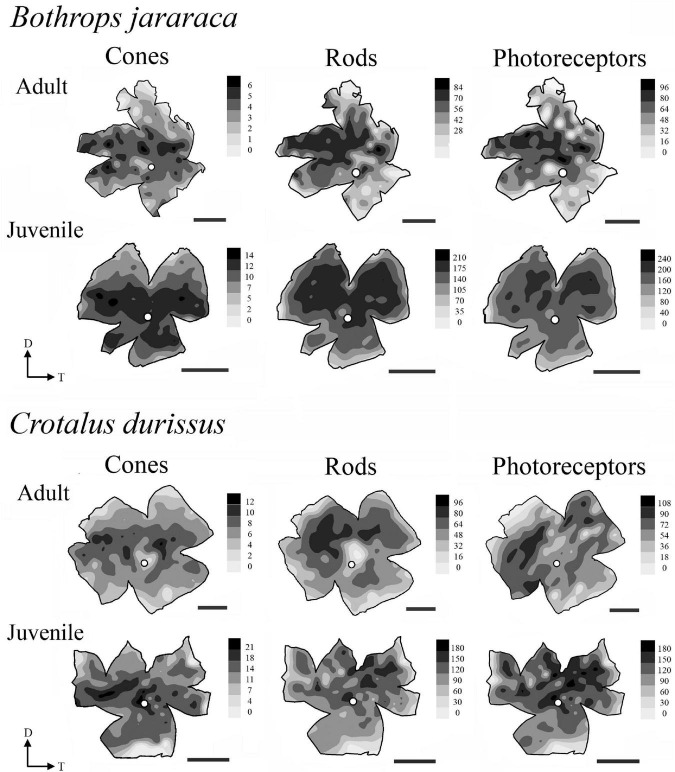
Representative topographic maps of the retinas of *B. jararaca* and *C. durissus*, showing the distribution of cones, rods, and photoreceptors in adults and juveniles. In adults of *B. jararaca*, both rods and cones form poorly defined visual streaks. In juveniles, rods are concentrated in a dorsal *area* and cones in a ventral *area*. In retinas of adults and juveniles of *C. durissus* rods are concentrated in anisotropic *area centralis* in the dorsal retina, and cones are arranged in poorly defined visual streaks. Gray bars indicate the density of cells per mm^2^, and the values should be multiplied by 10^3^. The optic nerve head is depicted as a white circle. D, dorsal; T, temporal. Scale bars 2 mm.

**FIGURE 8:**
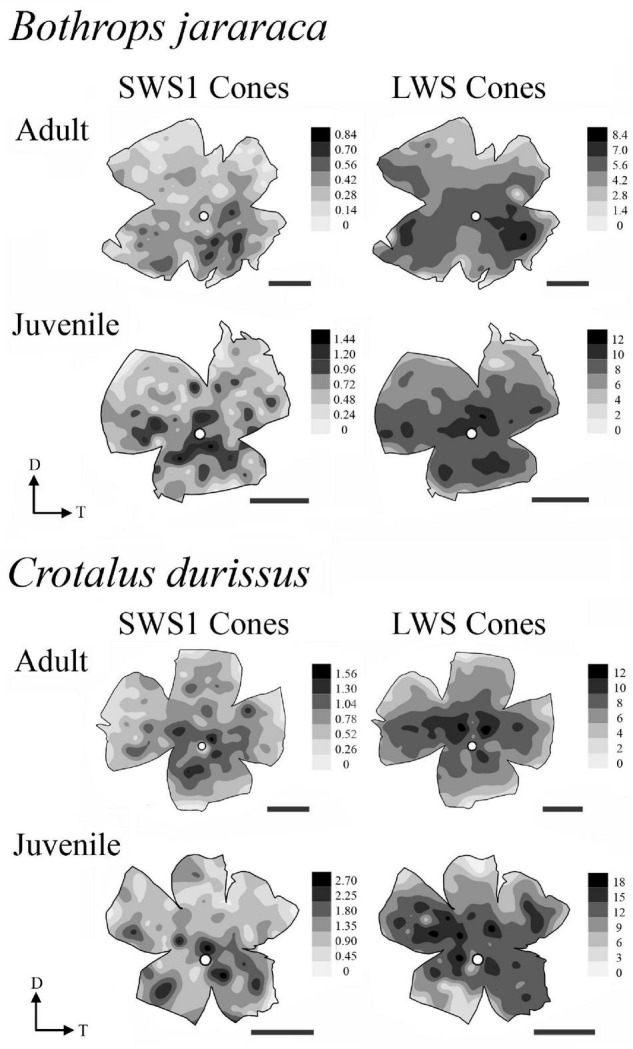
Representative retinal topographic maps of small single SWS1 cones and large single and double LWS cones of adults and juveniles of *B. jararaca* and *C. durissus*. Gray bars indicate the density of cells per mm^2^, and values should be multiplied by 10^3^. The optic nerve head is depicted as a white circle. D, dorsal; T, temporal. Scale bars 2 mm.

The distribution of photoreceptors showed differences between and within species. In adults of *B. jararaca*, rods and cones were distributed in poorly defined horizontal streaks (Figure 7 and Supplementary Figures 1, 2). In juveniles, rods were concentrated in an anisotropic *area centralis* in the dorsal retina, while cones were concentrated in the ventral retina (Figure 7 and Supplementary Figures 1, 2). Mean density values were estimated from retinal sectors (dorsal, ventral, temporal, and nasal). In adults and juveniles of *B. jararaca*, higher densities of rods were located in the temporal retina. Higher density of cones were located in the ventral retina of juveniles and in the temporal region in adults (Supplementary Figure 3 and Supplementary Table 4). In *C. durissus*, the isodensity maps of rods and cones were similar between adults and juveniles. Rods were concentrated in the dorsal retina in an anisotropic *area centralis*, and cones were organized in poorly defined visual streaks (Figure 7 and Supplementary Figures 1, 2). Density estimates of retinal sectors indicated higher density of rods in the temporal retina of juveniles and in the dorsal retina of adults. Cones had higher densities in the temporal region in adults and in the nasal region in juveniles (Supplementary Figure 3 and Supplementary Table 4). In both species, the SWS1 cones showed diffuse distributions, with peak densities located in the central or ventral retina (Figure 8 and Supplementary Figure 4). The distribution of LWS cones in the retinas of adults and juveniles of both species was similar to that observed for the distribution of total cones, with poorly defined visual streaks in adults of both species and in juveniles of *C. durissus*, and a higher concentration of LWS cones in the central and ventral retina of juveniles of *B. jararaca* (Figure 8 and Supplementary Figure 5).

### Ganglion Cell Layer Cells and Estimates of the Spatial Resolving Power

**FIGURE 9:**
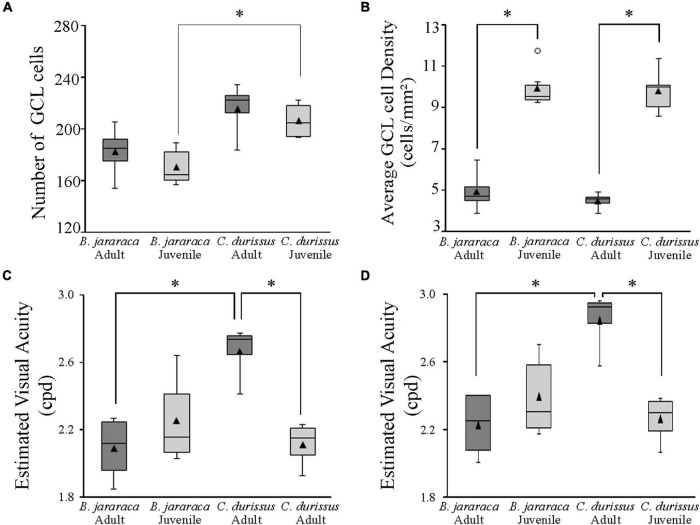
Boxplot representations of the medians (thick black lines) and quartiles (boxes) of **(A)** the total number of GCL cells and **(B)** the mean density of GCL cells in adults and juveniles of *B. jararaca* and *C. durissus*. The values in **(A,B)** should be multiplied by 10^3^. The open circle in **(B)** indicates an outlier value. Boxplots showing the estimated visual acuity considering **(C)** a square array of the ganglion cells, and **(D)** a hexagonal array of ganglion cells, in adults and juveniles of *B. jararaca* and *C. durissus*. The mean densities are represented by the triangles. Groups with statistically significant differences are indicated by asterisk (*p < 0.05).

**FIGURE 10:**
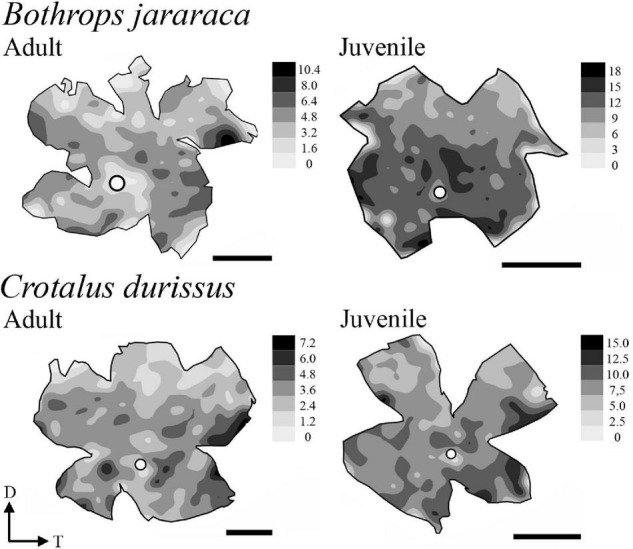
Representative retinal topographic maps of the GCL cells of adults and juveniles of *B. jararaca* and *C. durissus*. Gray bars indicate the density of cells per mm^2^, and the values should be multiplied by 10^3^. The optic nerve head is depicted as a white circle. D, dorsal; T, temporal. Scale bars 2 mm.

The population of GCL cells was estimated from 11 retinas of *B. jararaca* (adults: *n* = 4, juveniles: *n* = 7) and 9 retinas of *C. durissus* (adults: *n* = 4, juveniles: *n* = 5) (Table 4). The total population of GCL cells was similar between adults and juveniles of both species, ranging from 169,569 ± 13,730 (median: 163,733) cells in juveniles of *B. jararaca* and 215,141 ± 22,291 (median: 221,798) cells in adults of *C. durissus* (Figure 9 and Table 4). The average density of GCL cells was significantly higher in retinas of juveniles (9,871 ± 900 cells/mm^2^; median: 9,496 cells/mm^2^ in *B. jararaca* and 9,774 ± 1,078 cells/mm^2^; median: 9,951 cells/mm^2^ in *C. durissus*) compared with adults (4,880 ± 1,092 cells/mm^2^; median: 4,657 cells/mm^2^ in *B. jararaca* and 4,429 ± 448 cells/mm^2^; median: 4,517 cells/mm^2^ in *C. durissus*) (Figure 9 and Table 4). In the four groups analyzed, the isodensity maps of GCL cells did not show defined distribution patterns (Figure 10 and Supplementary Figure 6). In retinas of adults and juveniles of *C. durissus* and adults of *B. jararaca*, we observed diffuse distributions and peak density of cells in the temporal retina (Figure 10 and Supplementary Figure 6). In juveniles of *B. jararaca* the GCL cells were concentrated in the ventral retina with a decreasing ventral-dorsal gradient and peak density located in the ventral area (Figure 10 and Supplementary Figure 6). Density estimates in retinal sectors showed higher values in the temporal retina of adults and juveniles of *C. durissus* and adults of *B. jararaca* (Supplementary Figure 3 and Supplementary Table 5). In juveniles of *B. jararaca*, higher densities were located in the ventral retinas (Supplementary Figure 3 and Supplementary Table 5), in agreement with the observed isodensity maps (Figure 10 and Supplementary Figure 6).

**TABLE 4:** Stereological assessment of the population of GCL cells of adults and juveniles of *B. jararaca* and *C. durissus* and anatomical parameters used to estimate the upper limit of spatial resolution.

Species	Retinal area (mm^2^)	Total cells in the GCL	CE	Mean density (cells/mm^2^)	Peak density (cells/mm^2^)	Minimum density (cells/mm^2^)	asf	Eye axial length (mm)	PND mm	Spatial resolution (cpd)	
										Square array	Hexagonal array
***B. jararaca***											
Bj-A#1-RE	47.8	181,812	0.02	3,804	7,111	533	0.034	5.4	2.7	2.0	2.1
Bj-A#2-RE	39.6	187,201	0.04	4,680	10,489	356	0.029	5.0	2.5	2.3	2.4
Bj-A#4-RE	32.6	152,918	0.04	4,634	9,956	533	0.039	5.1	2.6	2.2	2.4
Bj-A#7-RE	31.8	204,869	0.01	6,402	10,311	533	0.044	4.2	2.1	1.8	2.0
Mean ± sd	38.0 ± 7.4	181,700 ± 21,568	0.03 ± 0.01	4,880 ± 1,092	9,467 ± 1,586	489 ± 89	0.037 ± 0.006	4.9 ± 0.5	2.5 ± 0.3	2.1 ± 0.2	2.2 ± 0.2
Bj-J#1-RE	16.9	157,709	0.03	9,331	17,956	356	0.062	3.2	1.2	2.6	2.7
Bj-J#5-LE	16.6	155,611	0.02	9,374	15,822	533	0.088	2.8	1.1	2.1	2.2
Bj-J#6-LE	19.1	188,770	0.03	9,883	15,487	889	0.060	3.4	1.3	2.4	2.6
Bj-J#7-RE	17.0	161,431	0.02	9,496	16,000	178	0.089	2.7	1.0	2.0	2.2
Bj-J#8-LE	15.5	187,434	0.02	11,715	18,133	533	0.087	3.0	1.1	2.4	2.5
Bj-J#9-LE	15.6	163,733	0.03	10,233	16,711	178	0.082	2.7	1.0	2.0	2.2
Bj-J#10-RE	19.3	172,298	0.02	9,068	14,933	533	0.062	3.0	1.1	2.1	2.3
Mean ± sd	17.1 ± 1.5	169,569 ± 13,730	0.02 ± 0.00	9,871 ± 900	16,435 ± 1,224	457 ± 248	0.076 ± 0.014	3.0 ± 0.2	1.1 ± 0.1	2.2 ± 0.2	2.4 ± 0.2
***C. durissus***											
Cd-A#3-RE	49.2	221,415	0.03	4,500	9,778	178	0.034	5.2	2.2	2.7	2.9
Cd-A#6-LE	37.5	182,861	0.03	4,876	9,778	356	0.034	5.3	2.2	2.8	3.0
Cd-A#7-RE	49.0	222,181	0.05	4,534	11,022	356	0.021	4.4	1.8	2.4	2.6
Cd-A#8-RE	61.5	234,109	0.03	3,807	7,289	711	0.018	6.1	2.6	2.7	2.9
Mean ± sd	49.3 ± 9.8	215,141 ± 22,291	0.03 ± 0.01	4,429 ± 448	9,467 ± 1,566	400 ± 224	0.027 ± 0.008	5.3 ± 0.7	2.2 ± 0.3	2.6 ± 0.2	2.8 ± 0.2
Cd-J#2-RE	19.6	222,188	0.02	11,336	16,356	2,133	0.060	3.3	1.4	2.2	2.4
Cd-J#3-RE	21.5	193,436	0.03	8,997	14,400	1,422	0.045	3.8	1.6	2.1	2.2
Cd-J#4-LE	21.9	217,938	0.03	9,951	16,178	2,489	0.045	−	−	−	−
Cd-J#7-LE	19.2	192,725	0.03	10,038	16,711	2,489	0.058	3.2	1.4	2.2	2.4
Cd-J#8-LE	23.9	204,274	0.03	8,547	13,156	1,067	0.043	3.2	1.3	1.9	2.1
Mean ± sd	21.2 ± 1.9	206,112 ± 13,616	0.03 ± 0.00	9,774 ± 1,078	15,360 ± 1,522	1,920 ± 646	0.050 ± 0.008	3.4 ± 0.3	1.4 ± 0.1	2.1 ± 0.1	2.3 ± 0.1

*sd, standard deviation; CE, Scheaffer’s coefficient of error; PND, posterior nodal distance; cpd, cycles per degree; RE, right eye; LE, left eye.*

The theoretical upper limits of spatial resolving power were estimated based on the ganglion cell peak densities using two approaches, one considering that the ganglion cells are organized in a hexagonal array and the other considering a square array. Adults and juveniles of *B. jararaca* had similar estimated visual acuity values, with 2.2 ± 0.2 cycles per degree (cpd) (median: 2.3 cpd) and 2.4 ± 0.2 cpd (median: 2.3 cpd), respectively (Figure 9 and Table 4). In *C. durissus* the estimated visual acuity was significantly higher in adults, with 2.8 ± 0.2 cpd (median: 2.9 cpd), compared to juveniles, with 2.3 ± 0.1 cpd (median: 2.3 cpd) (Figure 9 and Table 4). The estimated acuity values of adults of *C. durissus* were also significantly higher than of adults of *B. jararaca* (Figure 9 and Table 4).

From the estimated visual acuity values we calculated the minimum angle of resolution. The estimated distance relevant for predatory behavior was calculated considering the tangent of the minimum angle of resolution and minimum target size. For species with the lowest (2.2 cpd) and highest (2.8 cpd) spatial resolving power, we estimated a minimum angle of resolution of 0.45° and 0.36°. In an ecological context, snakes with lower estimated spatial resolution can observe an object with a minimum size of 10 cm, such as a small mammal, at a distance of approximately 13 m. Snakes with the higher presumed spatial resolution might be able to observe an object of the same size at a distance of about 17 m. These estimates indicate that at these predicted distances objects larger than the minimum target size can be spatially detected.

## Discussion

**FIGURE 11:**
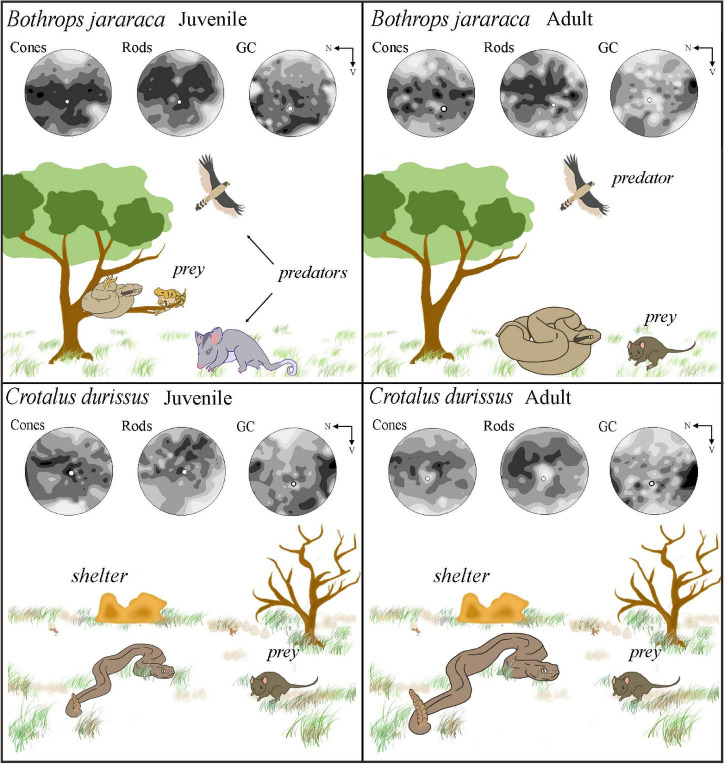
Schematic diagram illustrating the niche occupied by adults and juveniles of *Bothrops jararaca* and *Crotalus durissus*, and the distribution of neurons in the retinas of each species. **(Upper panel)** *B. jararaca* inhabits forested areas and has ontogenetic changes in diet, behavior, and microhabitat occupied. Juveniles are arboreal, feed on ectothermic prey (attracted by caudal luring), and are susceptible to predation pressure coming from different directions. A higher density of cones and GCL cells in the ventral retina might benefit the view of aerial predators under photopic conditions. A higher density of rods in the ventral retina might favor the view of terrestrial predators in the lower visual field under scotopic conditions. Adults are terrestrial and actively hunt for endothermic prey. Cones and rods are distributed in poorly defined visual streaks, and GCL cells have higher densities in the temporal retina. These specializations might benefit the panoramic view of the environment under photopic and scotopic conditions and of strike performances. **(Lower panel)** Adults and juveniles of *C. durissus* occupy open environments (Cerrado) and feed on endothermic prey. In both, cones form poorly defined visual streaks that might benefit scanning the environment to search for shelters for body temperature control during the day to avoid overheating, a usual behavior in snakes that occupy open areas. The higher density of rods in the dorsal retina might favor foraging behavior at scotopic conditions. The peak density of GCL cells in the temporal retina might benefit the view of the frontal field and favor strike performances. N, nasal; V, ventral.

In this study, we analyzed the density and distribution of neurons in whole-mounted retinas of Viperidae snakes, considering an ontogenetic approach. Our analyzes revealed a predominance of rods in the outer retinas of *Bothrops jararaca* and *Crotalus durissus*, and three distinct populations of cones: single and double cones containing the LWS photopigment and single cones containing the SWS1 photopigment, as previously described for viperids (Walls, 1942; Underwood, 1967b; Bittencourt et al., 2019; Gower et al., 2019). Our results showed that the two species have similar density values of photoreceptors and GCL cells. However, the distribution of these neurons differed between species and between juveniles and adults of *B. jararaca*, pointing to a reorganization of the retinal architecture that might be associated with the ontogenetic changes in the niche occupied and hunting strategies, as summarized in Figure 11.

### Photoreceptor Population

The retinas of *B. jararaca* and *C. durissus* had a high proportion of rods (about 90% of the photoreceptors) (Figure 5 and Table 2), which indicates high sensitivity to light, in agreement with their nocturnal or crepuscular activity pattern (Martins et al., 2001; Fiorillo et al., 2020b). Previous studies described the predominance of rods in retinas of viperid snakes based on analysis of retinal sections (Walls, 1934, 1942; Underwood, 1967b; Bittencourt et al., 2019; Gower et al., 2019) and fragments of flat-mounted retinas (Gower et al., 2019). Snakes stand out among vertebrates by their highly variable patterns of photoreceptor morphology (Walls, 1942). In the Caenophidia group (“advanced” snakes), nocturnal species from different families, including viperids, have four types of photoreceptors, with a unique type of double cone that differs from double cones of other vertebrates, with a large principal member and an extremely slender and attached accessory member (Walls, 1942). On the other hand, diurnal caenophidian snakes have “pure-cone” retinas, with the absence of typical rods, the presence of a transmuted cone-like rod, and lower photoreceptor density compared to nocturnal species (Walls, 1942; Schott et al., 2016; Hauzman et al., 2017; Hauzman, 2020).

The comparison of the populations of cones and rods in *B. jararaca* and *C. durissus* showed a higher proportion of cones in retinas of *C. durissus* (10% in juveniles and adults) compared to *B. jararaca* (7.5% in juveniles and 6.5% in adults) (Figure 5 and Table 2). This difference, although subtle, might reflect functional differences in temporal and spatial resolution between both species, which agree with the habitat occupied. *C. durissus* inhabit predominantly open areas of the Cerrado, with higher incidence of light (Miranda et al., 1997) compared to the closed forested areas predominantly occupied by *B. jararaca* (McWilliam et al., 1993; de Paula and Lemos Filho, 2001). In both species, the population of cones is dominated by large single cones and double cones that contain the LWS photopigment (about 80–90%) (Figure 6 and Table 3), with spectral sensitivity peak (λ_max_) predicted at 555 nm (Bittencourt et al., 2019). Small single cones with the SWS1 photopigment comprise about 10% of the cone population (Figure 6 and Table 3), with λ_max_ predicted at the UV range (360–370 nm) in both species (Bittencourt et al., 2019). Gower et al. (2019) identified double cones with the SWS1 photopigment in retinas of two viperid snakes, *Echis coloratus* and *C. durissus*, a unique type of photoreceptor described for the first time in vertebrates. However, in our analysis of whole-mounted retinas, this type of cone was not identified, and all double cones observed were found to contain only the LWS photopigment (Figure 2).

The distribution of photoreceptors differed between *C. durissus* and *B. jararaca* and between juveniles and adults of *B. jararaca* (Figure 7). In *C. durissus*, the visual streak formed by cones might reflect a better panoramic view of the environment under photopic conditions (Figure 7). This specialization might benefit scanning of the environment while searching for shelters for body temperature control during the day, a frequent behavior of snakes that occupy open environments where they are subject to overheating (Tozetti and Martins, 2008). On the other hand, the higher density of rods in the dorsal retina (Figure 7) might improve light sensitivity in the lower visual field, possibly favoring foraging behavior and searching for rodents during twilight and at night. These patterns of distribution of cones and rods were found in both, juveniles and adults of *C. durissus* (Figures 7, 11).

In *B. jararaca*, ontogenetic changes in the niche occupied seem to be associated with plasticity of the retinal architecture. In juveniles, a higher density of cones was observed in the ventral retina (Figures 7, 11), a specialization that might provide higher acuity in the upper visual field. Oppositely, a higher density of rods in the dorsal retina might benefit the view of the lower visual field under scotopic conditions (Figure 7). Juveniles of *B. jararaca* occupy the arboreal stratum and use sit-and-wait and caudal luring as hunting strategies. Therefore, it is plausible to speculate that the difference in the distribution of cones and rods might be associated with the direction of predation pressure. Cones located in a ventral *area* might favor the view of aerial predators, such as diurnal birds approaching from above during the day (Figure 11) (Sazima, 1992; Costa et al., 2014). Higher concentration of rods in the dorsal retina might benefit the view of terrestrial predators, such as marsupials, approaching from below, during the night (Figure 11) (Emmons, 1990; Sazima, 1992; Jared et al., 1998; Oliveira and Santori, 1999). Compared to terrestrial snakes, arboreal species display a higher number of defensive tactics, which is likely associated with greater exposure to predators approaching from a variety of directions (Martins et al., 2008).

Adults of *B. jararaca* occupy terrestrial environments, lose the caudal luring, and actively forage for endothermic prey (Sazima, 1992, 2006). Their retinas have cones and rods distributed in poorly defined visual streaks, a specialization that benefits the panoramic view of the terrestrial forested stratum under photopic and scotopic conditions (Figures 7, 11). A similar distribution of cones was described in terrestrial, arboreal and semiaquatic colubrids (Hauzman, 2014; Hauzman et al., 2014), indicating that this specialization is widely observed in snakes and might contribute to active foraging behavior in different environments. As far as we are aware, this is the first description of a horizontal streak formed by rods in the retinas of snakes.

The distribution of LWS cones (single and double) was similar to the distribution of total cones in both species, as expected based on their high proportion (Figure 8). The distribution of SWS1 cones did not show a defined pattern of specialization. Higher densities of SWS1 cones were found in the ventral and central retina in adults and juveniles of both species (Figure 8), as described for diurnal colubrid snakes (Hauzman et al., 2014). This distribution might favor the view of potential aerial predators approaching from the upper visual field, as many bird species are important predators of snakes (Martins and Oliveira, 1998; Tozetti, 2006; Specht et al., 2008; Costa et al., 2014). Higher densities of UV cones were also described in the ventral retina of mammals (Szél et al., 1992, 1994; Famiglietti and Sharpe, 1995; Peichl et al., 2005; Huber et al., 2010). In mice, UV cones contribute to chromatic discrimination of the upper visual field (Szatko et al., 2020), and might be relevant for visualizing the silhouette of aerial predators against the blue sky background (Calderone and Jacobs, 1995; Szél et al., 1992).

### Density and Topography of Ganglion Cell Layer Cells and Estimates of the Visual Acuity

The mean density of GCL cells was similar between *B. jararaca* and *C. durissus*. However, juveniles had higher average density values compared to adults (Figure 9 and Table 4). This difference can be attributed to the increase in the area of the retina. Juveniles have smaller retinas and thus, higher cell packaging. As the animals grow, the eye increases, such as the retinal area, but the cell population remains constant, resulting in lower density values. A higher GCL cell density in juveniles compared to adults was also described in fish (Hagedorn and Fernald, 1992; Bailes et al., 2006), birds (Straznicky and Chehade, 1987), amphibians (Nguyen and Straznicky, 1989), and mammals (Robinson et al., 1989). Considering there is no neuron loss or additional generation of cells in the GCL, we suggest the increase of retinal area followed by the decrease of cell density is associated with interstitial growth, as described for chicken retinas (Straznicky and Chehade, 1987). It is notable that in *Bothrops jararaca* these changes in cell density are associated with rearrangements of the cell distribution.

The distribution of GCL cells was similar between adults and juveniles of *C. durissus*, with no defined type of specialization, and with peak density of cells in the temporal retina (Figures 10, 11). This specialization might benefit the view of the frontal field and favor strike performance of endothermic prey. The same topographic pattern of GCL cells was found in retinas of adults of *B. jararaca* (Figures 10, 11). The visual information arriving in the midbrain, from the projections of this temporal specialization might be combined with infrared information from the loreal pit, a thermosensory organ of Crotalinae snakes located between the eyes and the nostrils (Noble and Schmidt, 1973). The nerve terminals of the loreal pit cells project to the optic tectum in the midbrain (Newman and Hartline, 1982), where the visual and thermosensory inputs are combined, allowing an integrated perception from ultra-violet to infrared wavelengths (Newman and Hartline, 1981; Moiseenkova et al., 2003; Goris, 2011), important in both scotopic and photopic conditions. During the night, thermal detection enables the perception of temperature changes. During the day, discrimination of infrared information may be compromised due to higher temperatures (Chen et al., 2012), yielding predominance of perceptual information to visual inputs.

In juveniles of *B. jararaca* the higher density of GCL cells in the ventral retina is in agreement with the distribution of cones (Figures 8, 10, 11), which indicates a higher convergence from cones to GCs in this region (from approximately 4:1 in the periphery, to ∼1:1 in the *area centralis*), enabling higher spatial resolution in the upper visual field. A similar distribution pattern of GCL cells, with higher density in the ventral retina was observed in the dipsadid snakes *Dipsas albifrons* and *Sibynomorphus neuwiedi* (Hauzman et al., 2018). Both species are nocturnal, semi-arboreal, and feed on ectothermic prey (goo-eaters) (Maia et al., 2011; Sazima and Muscat, 2016), such as juveniles of *B. jararaca*. Thus, we suggest that this type of specialization might be associated with the niche occupied by snakes, the direction of predatory threats, and particularities of hunting strategies, which do not involve active foraging of fast moving prey (Figure 11). Reorganization of the distribution of GCL cells according to ontogenetic changes in ecology and behavior was also described in frogs (Dunlop and Beazley, 1981) and in fish (Shand, 1997; Bozzano and Catalan, 2002; Miyazaki et al., 2011). In the fish *Acanthopagrus butcheri*, the retinas of juveniles have a temporal *area centralis*, which favors object detection in the frontal visual field and might be important for feeding on plankton in pelagic waters. On the other hand, adults occupy the benthic environment. This ecological switch is accompanied by a displacement of the *area* toward the dorsal or dorso-temporal retina, which might increase acuity in the lower-frontal visual field and benefit their hunting strategies for the capture of small fish, polychaete worms and detritus obtained from the benthos (Shand et al., 2000).

The estimated upper limits of spatial resolving power based on the peak cell density of GCL cells of the viperid snakes varied from 2 to 3 cpd (Table 4). These values were similar to those estimated for diurnal dipsadid snakes of the genus *Philodryas* spp. (Hauzman et al., 2014) and for the marine elapid *Aipysurus laevis* (Hart et al., 2012), with spatial resolution between 2.3 and 2.6 cpd, and were higher than the values estimated for nocturnal dipsadids, with about 1.3 cpd (Hauzman et al., 2018). Our comparisons showed slightly higher estimated visual acuity in adults of *C. durissus* compared to the other groups analyzed (Figure 9). We predicted that the estimated visual acuity of ∼2.3 cpd of adults and juveniles of *B. jararaca* and juveniles of *C. durissus* would allow the view of potential prey such as rodents, at a minimum size of 10 cm, at a distance of about 13 m. On the other hand, adults of *C. durissus* with estimated visual acuity at ∼2.8 cpd, might be able to detect the same minimum object size at a higher distance, about 17 m. These estimates also indicate that these snakes can have a high capability to observe potential predators such as birds of prey (Martins and Oliveira, 1998; Tozetti, 2006; Specht et al., 2008; Costa et al., 2014) at longer distances. However, behavioral analyzes are necessary to confirm if the estimated values of spatial resolution based on anatomical data are reached, and to ascertain whether the differences imply functional significance, relevant to the species ecology and visual behavior.

## Conclusion

Interspecific differences in the density and distribution of retinal neurons were identified in the viperid snakes *B. jararaca* and *C. durissus*. Rod-dominated retinas represent a functional advantage associated with the nocturnal activity pattern of both species. The higher proportion of cones in *C. durissus* might represent an adaptation to the open and brighter Cerrado environment. This study showed for the first time in snakes that ontogenetic changes in ecology and behavior are associated with morphological plasticity of the retinas. We suggest that the differences in the niche occupied, hunting strategies, prey type, and direction of approach of predatory threats are reflected in reorganizations of the distribution of retinal neurons between juveniles and adults of *B. jararaca*. In comparison, the absence of variation in neuron distribution patterns in retinas of *C. durissus* throughout life agrees with the maintenance of the same ecological and behavioral traits in juveniles and adults. These results highlight the importance of retinal specializations for the performance of visually guided behaviors, and how habitat use and hunting strategies might represent relevant ecological forces that shape the retinal architecture in snakes. Future behavioral studies should be applied to verify functional implications of the retinal organizations as well as the estimated upper limits of spatial resolving power, between 2 and 3 cpd. In addition, the patterns of neuronal connections in the retina rely on a highly intricate network for visual processing. The results of this study open up a new avenue for future analysis on the connectivity patterns of the inner retina of Viperidae snakes.

## Data Availability Statement

The original contributions presented in the study are included in the article/Supplementary Material, further inquiries can be directed to the corresponding author/s.

## Ethics Statement

The animal study was reviewed and approved by the Ethics Committee of Animal Research of the Psychology Institute, University of São Paulo, Brazil.

## Author Contributions

EH conceived the study. DV obtained the funding. JT and EH performed the research and wrote the manuscript. JT, EH, and DV analyzed the data. All authors contributed to manuscript revision and approved the submitted version.

## Conflict of Interest

The authors declare that the research was conducted in the absence of any commercial or financial relationships that could be construed as a potential conflict of interest.

## Publisher’s Note

All claims expressed in this article are solely those of the authors and do not necessarily represent those of their affiliated organizations, or those of the publisher, the editors and the reviewers. Any product that may be evaluated in this article, or claim that may be made by its manufacturer, is not guaranteed or endorsed by the publisher.

## Footnotes

1
https://cran.r-project.org/
