# Supplementary material for: Morphological Plasticity of the Retina of Viperidae Snakes Is Associated With Ontogenetic Changes in Ecology and Behavior
Source: Front Neuroanat. 2022 Jan 26;15:770804. doi: 10.3389/fnana.2021.770804 (PMC8825375; doi:10.3389/fnana.2021.770804)
Supplement: Supplementary file 1 [file Data_Sheet_1.pdf]

Supplementary Material

Morphological plasticity of the retina of Viperidae snakes is associated with ontogenetic changes in ecology and behavior.

Juliana H. Tashiro, Dora F. Ventura, Einat Hauzman

1. Supplementary Tables

Supplementary Table 1. Morphometric and ocular data from *B. jararaca* and *C. durissus.*

Species	Sex	Mass (g)	SVL (mm)	Eye Diameter (mm) RE	Eye Diameter (mm) LE	Length Diameter (mm) RE	Length Diameter (mm) LE	Retinal area (mm²) RE	Retinal area (mm²) LE
B. jararaca									
Bj-A#1	M	458.6	1170.0	5.4	5.5	2.8	-	47.8	-
Bj-A#2	F	442.6	1098.5	5.0	5.1	2.9	3.3	39.6	-
Bj-A#3	F	590.0	1060.0	5.2	5.4	2.8	3.2	46.7	-
Bj-A#4	F	381.2	1010.0	5.1	5.3	3.0	3.2	32.6	31.2
Bj-A#5	F	431.4	995.0	5.3	5.5	3.3	3.4	53.2	-
Bj-A#6	F	260.8	991.1	5.3	5.1	3.3	3.6	-	54.0
Bj-A#7	M	-	775.0	4.1	4.8	2.6	-	31.8	-
Mean ± sd		427.4±107.3	1014.2±123.6	5.1±0.4	5.2±0.3	3.0±0.3	3.3±0.2	42.0±8.7	42.6±16.1
Bj-J#1	F	16.9	368.0	3.2	3.4	2.0	-	16.9	
Bj-J#2	F	9.4	340.0	3.7	3.3	1.8	1.8	21.2	-
Bj-J#3	M	-	325.0	3.5	3.6	2.0	1.9	23.8	-
Bj-J#4	F	11.3	313.0	3.5	3.4	1.9	1.9	-	24.4
Bj-J#5	F	9.7	310.0	3.3	2.8	1.6	1.7	-	16.6
Bj-J#6	F	6.8	263.0	3.9	3.4	-	1.9	-	19.1
Bj-J#7	M	9.5	250.0	2.7	2.9	1.6	1.8	17.0	-
Bj-J#8	F	6.0	245.0	3.1	3.0	1.6	1.6	-	15.5
Bj-J#9	M	8.1	232.0	2.5	2.7	-	1.7	-	15.6
Bj-J#10	F	7.1	230.0	-	3.0	1.8	1.8	19.3	-
Mean ± sd		9.4±3.3	287.6±49.4	3.3±0.4	3.2±0.3	1.8±0.2	1.8±0.1	19.6±2.9	18.2±3.7
C. durissus									
Cd-A#1	M	734.4	1230.0	5.6	5.3	-	3.5	-	55.1
Cd-A#2	M	570.0	945.0	5.4	5.1	3.1	2.9	49.7	-
Cd-A#3	F	570.0	886.0	5.2	5.2	2.7	2.5	49.2	48.3
Cd-A#4	M	520.1	840.0	4.6	4.8	2.7	2.7	-	41.8
Cd-A#5	F	417.8	765.0	4.8	4.9	2.7	2.7	39.5	-
Cd-A#6	M	484.6	760.0	5,7	5.3	-	2.9	-	37.5
Cd-A#7	M	390.0	760.0	4.4	4.5	2.6	2.4	49.0	-
Cd-A#8	M	-	-	6.1	6.1	3.8	3.8	61.5	-
Mean ± sd		526.6±114.9	883.7±168.5	5.2±0.6	5.2±0.5	2.9±0.5	2.9±0.5	49.8±7.8	45.7±7.7
Cd-J#1	M	20.0	370.0	3.7	3.3	2.7	1.8	23.3	-
Cd-J#2	F	21.0	360.0	3.3	-	1.6	-	19.6	-
Cd-J#3	M	27.0	330.0	3.8	3.7	1.7	1.7	21.5	-
Cd-J#4	M	23.0	330.0	-	-	-	1.7	-	21.9
Cd-J#5	F	25.0	320.0	3.5	3.5	1.5	1.5	22.4	-
Cd-J#6	F	25.0	310.5	3.7	3.3	1.8	1.7	20.3	25.0
Cd-J#7	M	22.0	300.0	3.9	3.2	-	1.7	-	19.2
Cd-J#8	F	25.0	310.0	3.7	3.2	-	1.7	-	23.9
Mean ± sd		23.5±2.4	328.1±24.7	3.7±0.2	3.4±0.2	1.9±0.5	1.7±0.1	21.4±1.5	22.5±2.5

sd, standard deviation; g, grams; SVL, snout-vent length; RE, right eye; LE, left eye; mm, millimeter; F, female; M, male.

Supplementary Table 2. Stereological parameters used to estimate the number and distribution of total photoreceptors, SWS1 and LWS cones, in adults and juveniles of *B. jararaca* and *C. durissus.*

Species	Retinal area (mm²)	Total Photoreceptors			SWS1 cones			LWS cones		
		Counting frame	Grid	Objective/ NA	Counting frame	Grid	Objective/ NA	Counting frame	Grid	Objective/ NA
B. jararaca										
Bj-A#3-RE	46.7	50x50	500x500	100x/NA1.4-0.7oil	180x180	500x500	40x/NA0.80	180x180	500x500	40x/NA0.80
Bj-A#4-LE	31.2	50x50	400x400	100x/NA1.4-0.7oil	-	-	-	150x150	400x400	40x/NA0.80
Bj-A#5-RE	53.2	50x50	500x500	100x/NA1.4-0.7oil	180x180	500x500	40x/NA0.80	180x180	500x500	40x/NA0.80
Bj-A#6-LE	54.0	50x50	500x500	100x/NA1.4-0.7oil	180x180	550x550	40x/NA0.80	-	-	-
Mean ± sd	46.3 ± 10.6									
Bj-J#2-RE	21.2	50x50	350x350	100x/NA1.4-0.7oil	100x100	350x350	40x/NA0.80	100x100	350x350	40x/NA0.80
Bj-J#3-RE	23.8	50x50	350x350	100x/NA1.4-0.7oil	100x100	350x350	40x/NA0.80	100x100	350x350	40x/NA0.80
Bj-J#4-LE	24.4	50x50	350x350	100x/NA1.4-0.7oil	100x100	350x350	40x/NA0.80	100x100	350x350	40x/NA0.80
Mean ± sd	23.1 ± 1.7									
C. durissus										
Cd-A#1-LE	55.1	50x50	500x500	100x/NA1.4-0.7oil	180x180	550x550	40x/NA0.80	-	-	-
Cd-A#2-RE	49.7	50x50	500x500	100x/NA1.4-0.7oil	-	-	-	180x180	500x500	40x/NA0.80
Cd-A#3-LE	48.3	50x50	500x500	100x/NA1.4-0.7oil	-	-	-	180x180	500x500	40x/NA0.80
Cd-A#4-LE	41.8	50x50	450x450	100x/NA1.4-0.7oil	180x180	450x450	40x/NA0.80	180x180	450x450	40x/NA0.80
Cd-A#5-RE	39.5	50x50	450x450	100x/NA1.4-0.7oil	180x180	450x450	40x/NA0.80	180x180	450x450	40x/NA0.80
Mean ± sd	46.9 ± 6.3									
Cd-J#1-RE	23.3	50x50	350x350	100x/NA1.4-0.7oil	100x100	350x350	40x/NA0.80	100x100	350x350	40x/NA0.80
Cd-J#5-RE	22.4	50x50	350x350	100x/NA1.4-0.7oil	100x100	350x350	40x/NA0.80	100x100	350x360	40x/NA0.80
Cd-J#6-LE	25.0	50x50	350x350	100x/NA1.4-0.7oil	100x100	350x350	40x/NA0.80	100x100	350x350	40x/NA0.80
Mean ± sd	23.6 ± 1.3									

sd, standard deviation; RE, right eye; LE, left eye.

Supplementary Table 3. Stereological parameters used to estimate the number and distribution of GCL cells in adults and juveniles of *B. jararaca* and *C. durissus*.

Species	Retinal area (mm²)	Counting frame	Grid	Objective/ NA
B. jararaca				
Bj-A#1-RE	47.8	75x75	400x400	63x/NA 1.4-0.60 oil
Bj-A#2-RE	39.6	75x75	400x400	63x/NA 1.4-0.60 oil
Bj-A#4-RE	32.6	75x75	350x350	63x/NA 1.4-0.60 oil
Bj-A#7-RE	31.8	75x75	350x350	63x/NA 1.4-0.60 oil
Mean ± sd	38.0 ± 7.4			
Bj-J#1-RE	16.9	75x75	300x300	63x/NA 1.4-0.60 oil
Bj-J#5-LE	16.6	75x75	250x250	63x/NA 1.4-0.60 oil
Bj-J#6-LE	19.1	75x75	300x300	63x/NA 1.4-0.60 oil
Bj-J#7-RE	17.0	75x75	250x250	63x/NA 1.4-0.60 oil
Bj-J#8-LE	15.5	75x75	250x250	63x/NA 1.4-0.60 oil
Bj-J#9-LE	15.6	75x75	250x250	63x/NA 1.4-0.60 oil
Bj-J#10-RE	19.3	75x75	300x300	63x/NA 1.4-0.60 oil
Mean ± sd	17.1 ± 1.5			
C. durissus				
Cd-A#3-RE	49.2	75x75	400x400	63x/NA 1.4-0.60 oil
Cd-A#6-LE	37.5	75x75	400x400	63x/NA 1.4-0.60 oil
Cd-A#7-RE	49.0	75x75	450x450	63x/NA 1.4-0.60 oil
Cd-A#8-RE	61.5	75x75	550x550	63x/NA 1.4-0.60 oil
Mean ± sd	49.3 ± 9.8			
Cd-J#2-RE	19.6	75x75	300x300	63x/NA 1.4-0.60 oil
Cd-J#3-RE	21.5	75x75	350x350	63x/NA 1.4-0.60 oil
Cd-J#4-LE	21.9	75x75	350x350	63x/NA 1.4-0.60 oil
Cd-J#7-LE	19.2	75x75	300x300	63x/NA 1.4-0.60 oil
Cd-J#8-LE	23.9	75x75	350x350	63x/NA 1.4-0.60 oil
Mean ± sd	21.2 ± 1.9			

sd, standard deviation; RE, right eye; LE, left eye.

Supplementary Table 4. Mean densities from retinal subregions (dorsal, temporal, ventral and nasal) of cones and rods, in adults and juveniles of *B. jararaca* and *C. durissus.*

Species	Rods				Cones			
	Dorsal	Temporal	Ventral	Nasal	Dorsal	Temporal	Ventral	Nasal
B. jararaca								
Bj-A#3-RE	72,144.7	77,838.5	64,600.0	71,140.4	4,578.7	6,432.0	5,482.4	6,093.6
Bj-A#4-LE	90,237.0	93,337.5	86,615.4	77,525.9	4,412.5	5,773.3	5,109.1	4,746.7
Bj-A#5-RE	66,736.0	73,095.1	61,113.5	62,925.5	3,897.9	5,544.8	5,232.4	4,855.2
Bj-A#6-LE	54,026.7	48,434.0	42,421.6	62,731.9	3,089.4	4,075.5	3,687.5	4,434.8
Mean ± sd	70,786.1 ± 15,027.4	73,176.3 ± 18,622.5	63,687.6 ± 18,123.5	68,580.9 ± 7,135.8	3,994.6 ± 669.5	5,456.4 ± 994.5	4,877.8 ± 808.6	5,032.6 ± 729.5
Bj-J#2-RE	162,400.0	166,769.2	140,246.2	162,541.2	8,308.6	11,620.0	11,502.2	11,528.9
Bj-J#3-RE	115,626.7	129,278.3	122,938.2	109,449.1	7,340.0	8,850.0	10,081.8	9,215.1
Bj-J#4-LE	112,658.1	110,960.0	100,807.4	109,983.7	8,916.7	11,572.9	11,269.2	9,586.4
Mean ± sd	130,228.2 ± 27,901.1	135,669.2 ± 28,448.2	121,330.6 ± 19,768.5	127,324.6 ± 30,499.6	8,188.4 ± 795.2	10,681.0 ± 1,585.8	10,951.1 ± 761.8	10,110.1 ± 1,242.6
C. durissus								
Cd-A#1-LE	59,947.8	58,792.3	52,349.1	64,938.2	5,460.9	6,307.7	6,092.9	6,414.5
Cd-A#2-RE	69,344.4	57,672.7	51,016.2	65,347.8	6,555.6	7,645.2	5,979.5	8,200.0
Cd-A#3-LE	73,245.7	69,243.5	59,760.0	69,981.8	7,176.5	8,100.0	7,317.6	7,787.2
Cd-A#4-LE	84,702.0	76,761.9	70,516.7	80,057.1	8,127.3	9,557.4	8,736.4	8,891.8
Cd-A#5-RE	77,229.3	87,989.5	70,181.8	79,114.3	7,117.6	10,241.9	8,481.8	8,684.7
Mean ± sd	72,893.9 ± 9,199.9	70,092.0 ± 12,722.5	60,764.8 ± 9,362.8	71,887.9 ± 7,308.6	6,887.6 ± 977.1	8,370.4 ± 1,562.5	7,321.6 ± 1,290.2	7,995.7 ± 982.7
Cd-J#1-RE	133,810.5	137,449.1	129,266.7	128,313.0	12,775.8	16,519.3	14,609.5	15,325.0
Cd-J#5-RE	102,075.0	116,617.4	106,284.4	118,691.7	11,769.7	14,409.3	13,493.3	15,600.0
Cd-J#6-LE	126,137.9	113,258.1	94,563.6	110,342.9	13,022.2	14,030.2	12,868.1	14,984.0
Mean ± sd	120,674.5 ± 16,558.2	122,441.5 ± 13,105.0	110,038.2 ± 17,653.4	119,115.9 ± 8,992.6	12,522.6 ± 977.1	14,986.3 ± 1,562.5	13,657.0 ± 1,290.2	15,303.0 ± 982.7

sd, standard deviation.

Supplementary Table 5. Mean densities from retinal subregions (dorsal, temporal, ventral and nasal) of GCL cells, in adults and juveniles of *B. jararaca* and *C. durissus*.

Species	GCL cells			
	Dorsal	Temporal	Ventral	Nasal
B. jararaca				
Bj-A#1-RE	3,167.4	3,954.4	3,885.3	4,183.9
Bj-A#2-RE	3,794.3	5,219.3	4,913.8	4,537.4
Bj-A#4-RE	4,119.8	5,167.0	4,146.8	5,015.4
Bj-A#7-RE	5,431.4	6,669.1	6,507.8	6,930.3
Mean±sd	4,128.2±954.4	5,252.5±1,110.6	4,863.4±1,179.9	5,166.7±1,224.0
Bj-J#1-RE	8,307.1	10,793.7	9,750.0	8,074.9
Bj-J#5-LE	7,971.6	9,009.9	10,009.6	9,849.3
Bj-J#6-LE	7,708.3	10,634.3	10,969.9	9,681.4
Bj-J#7-RE	7,446.9	9,158.4	10,664.5	10,356.3
Bj-J#8-LE	9,972.3	11,056.2	13,270.6	12,484.7
Bj-J#9-LE	8,672.8	10,778.6	10,337.3	10,951.1
Bj-J#10-RE	6,963.5	9,339.5	10,718.8	9,070.6
Mean±sd	8,148.9±978.8	10,110.1±893.9	10,817.2±1,161.6	10,066.8±1,406.4
C. durissus				
Cd-A#3-RE	3,409.3	5,201.8	4,715.0	4,517.9
Cd-A#6-LE	3,555.6	5,848.0	5,051.9	4,907.7
Cd-A#7-RE	3,744.9	6,008.9	4,983.3	3,374.3
Cd-A#8-RE	2,879.0	4,063.0	4,073.8	3,942.2
Mean±sd	3,397.2±371.8	5,280.4±883.4	4,706.0±445.8	4,185.5±670.6
Cd-J#2-RE	9,876.5	11,994.4	11,210.3	11,963.0
Cd-J#3-RE	7,562.8	9,600.0	9,759.5	8,956.0
Cd-J#4-LE	8,733.8	10,865.1	10,175.4	10,152.2
Cd-J#7-LE	9,422.2	10,907.7	10,427.9	9,182.8
Cd-J#8-LE	7,832.2	9,234.8	9,866.7	8,475.2
Mean±sd	8,685.5±994.0	10,520.4±1,111.5	10,288.0±578.7	9,745.8±1,381.7

sd, standard deviation.

2. Supplementary Figures

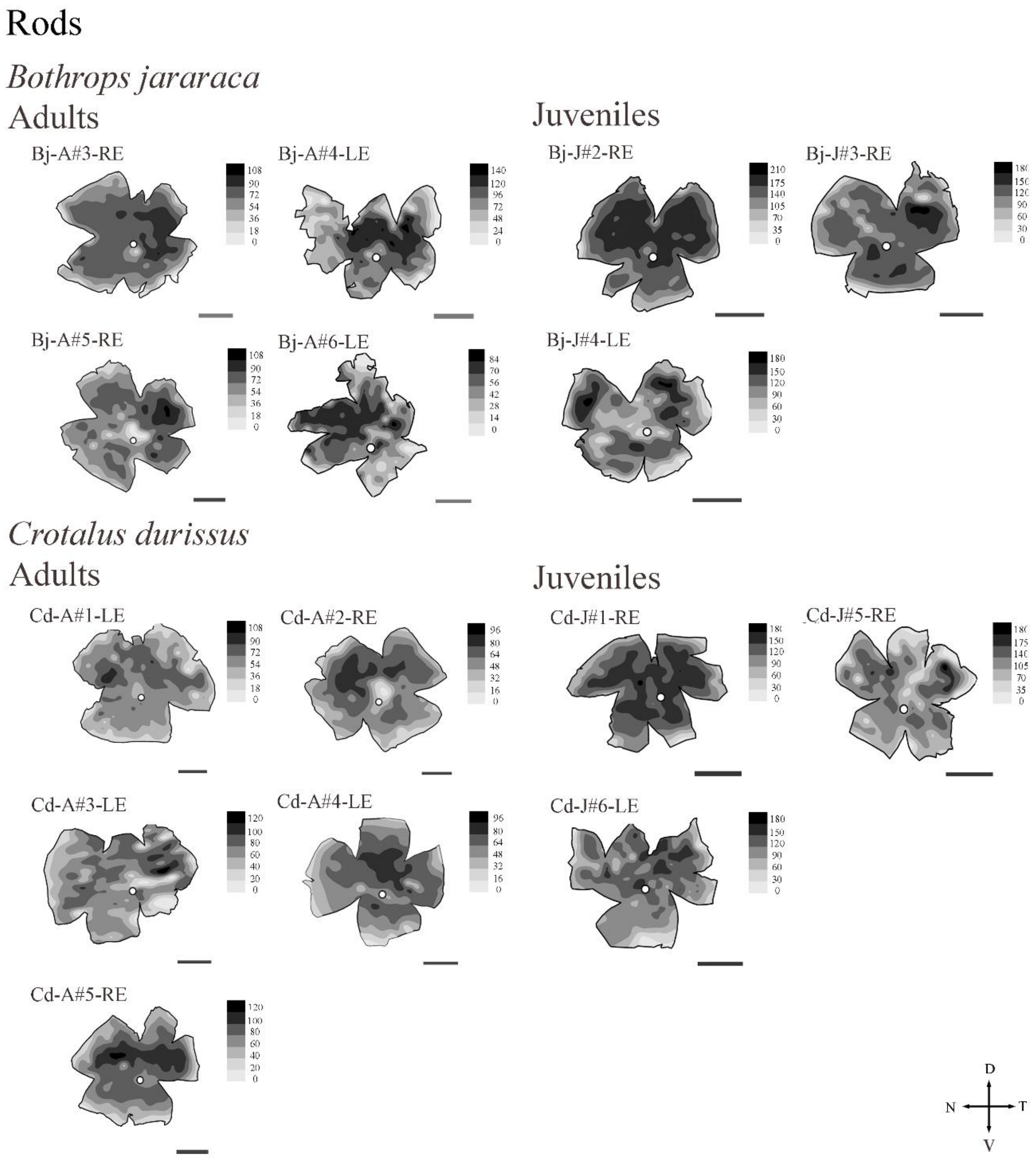

Supplementary Figure 1. Retinal topographic maps of rods of *B. jararaca* and *C. durissus*. In retinas of adults of *B. jararaca*, poorly defined visual streaks can be observed (Bj-A#4-LE, Bj-A#6-LE) or the absence of a defined distribution pattern, with rods concentrated in the dorsal-temporal retina (Bj-A#3-RE, Bj-A#5-RE). In juveniles of *B. jararaca*, rods are concentrated in the dorsal retina, as in adults and juveniles of *C. durissus*. Gray bars indicate the density of cells per mm², and the values should be multiplied by 10³. The optic nerve head is depicted as a white circle. D, dorsal; T, temporal; N, Nasal; V, Ventral. Scale bars 2 mm.

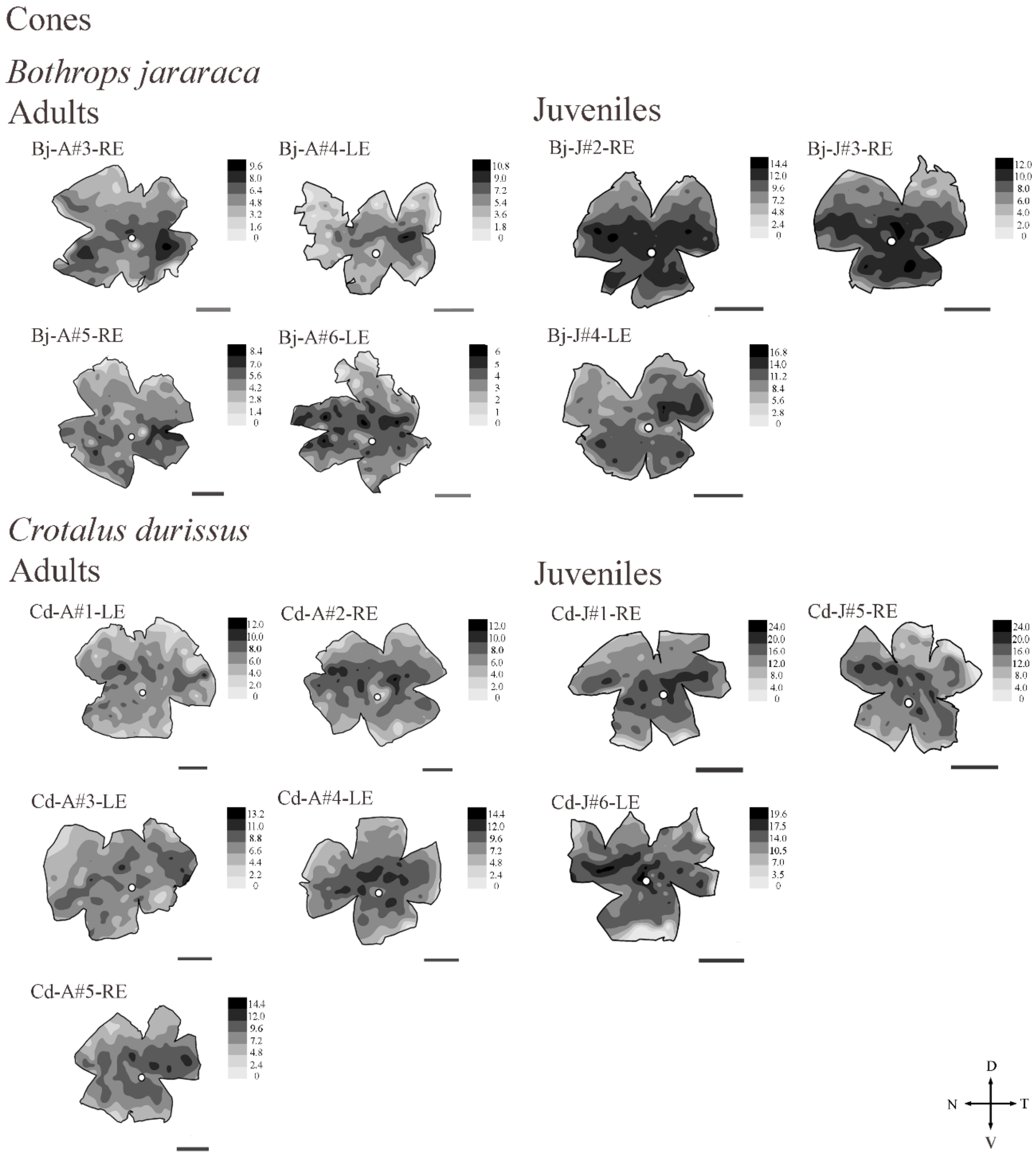

Supplementary Figure 2. Topographic maps of cones in retinas of *B. jararaca* and *C. durissus*. In adults of *B. jararaca*, cones form poorly defined visual streaks (Bj-A#3-RE, Bj-A#4-LE, Bj-A#6-LE) or are concentrated in the ventral-temporal retina (Bj-A#5-RE). In juveniles, cones are concentrated in the central-ventral retina. In retinas of adults and juveniles of *C. durissus* cones are arranged in poorly defined visual streaks. Gray bars indicate the density of cells per mm², and the values should be multiplied by 10^3. The optic nerve head is depicted as a white circle. D, dorsal; T, temporal; N, Nasal; V, Ventral. Scale bars 2 mm.

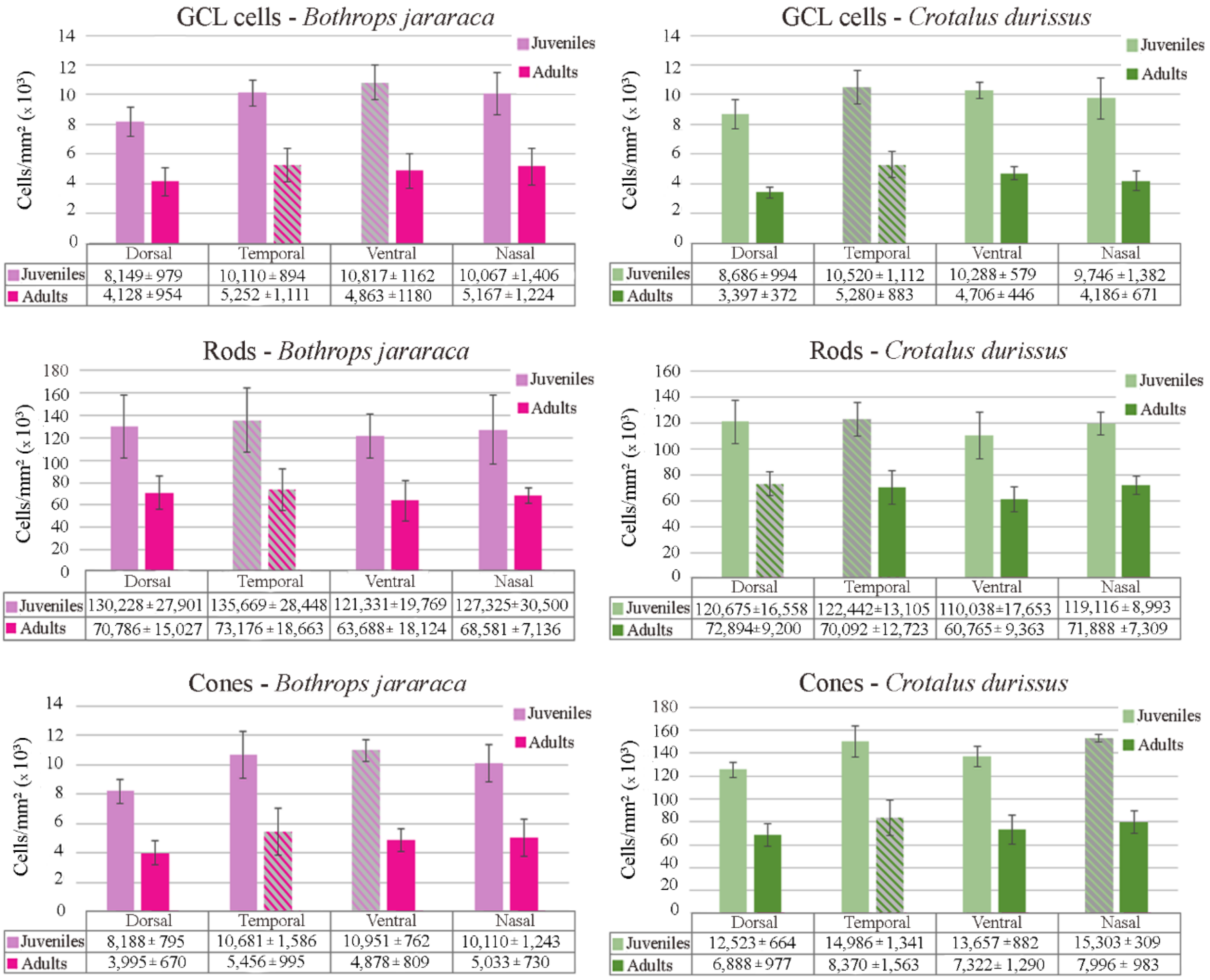

Supplementary Figure 3. Bar charts showing the mean density of GCL cells, rods and cones in retinal subregions (dorsal, temporal, ventral and nasal) in adults and juveniles of *B. jararaca* and *C. durissus*. The striped bars represent the subregions with higher mean density values.

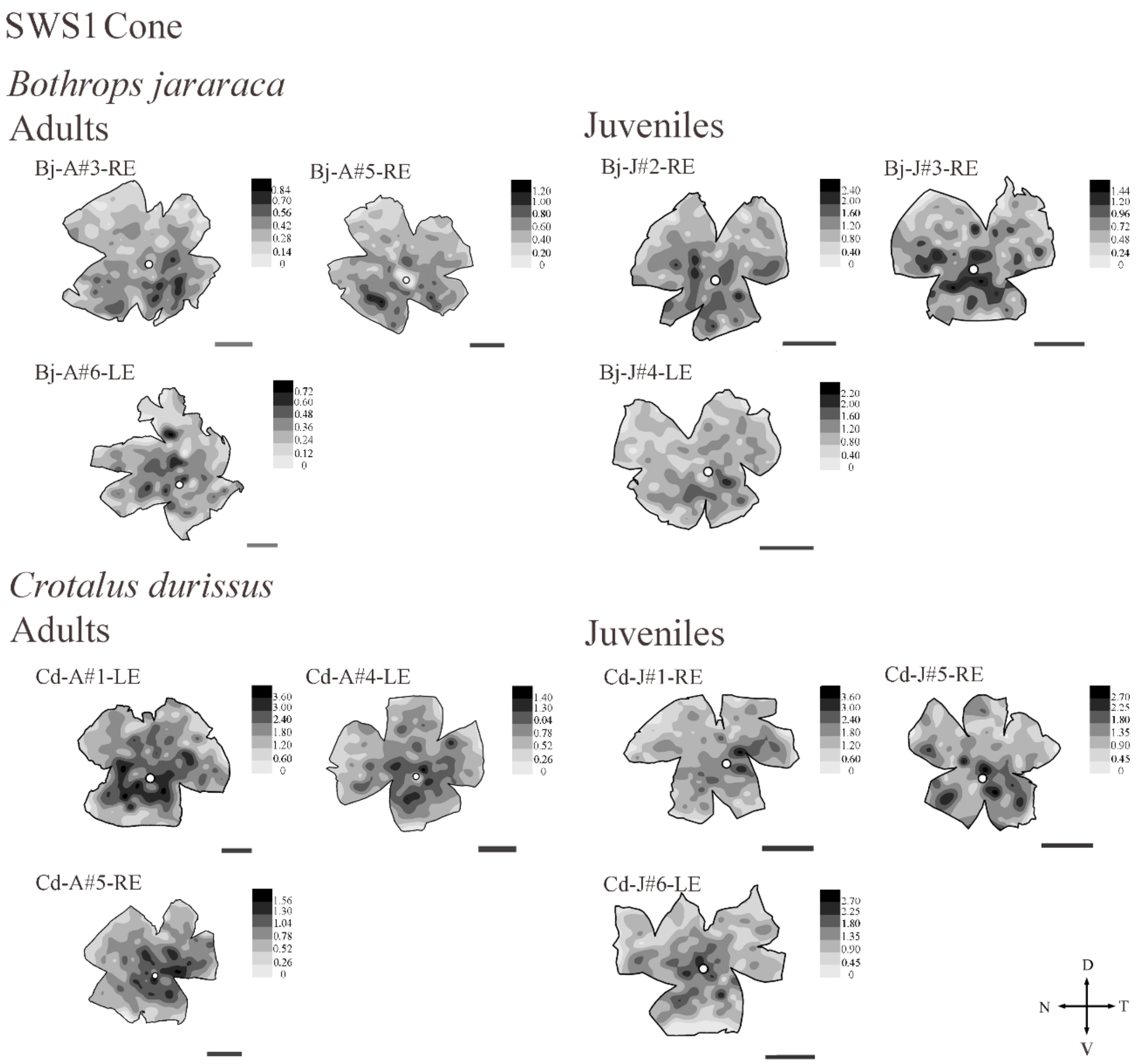

Supplementary Figure 4. Retinal topographic maps of SWS1 cones of adults and juveniles of *B. jararaca* and *C. durissus.* In all groups, the SWS1 cones do not assume a defined pattern of distribution (i.e. visual streak or *area centralis*). Higher densities are located in the ventral or central areas of the retinas. Gray bars indicate the density of cells per mm², and the values should be multiplied by 10^3. The optic nerve head is depicted as a white circle. D, dorsal; T, temporal; N, Nasal; V, Ventral. Scale bars 2 mm.

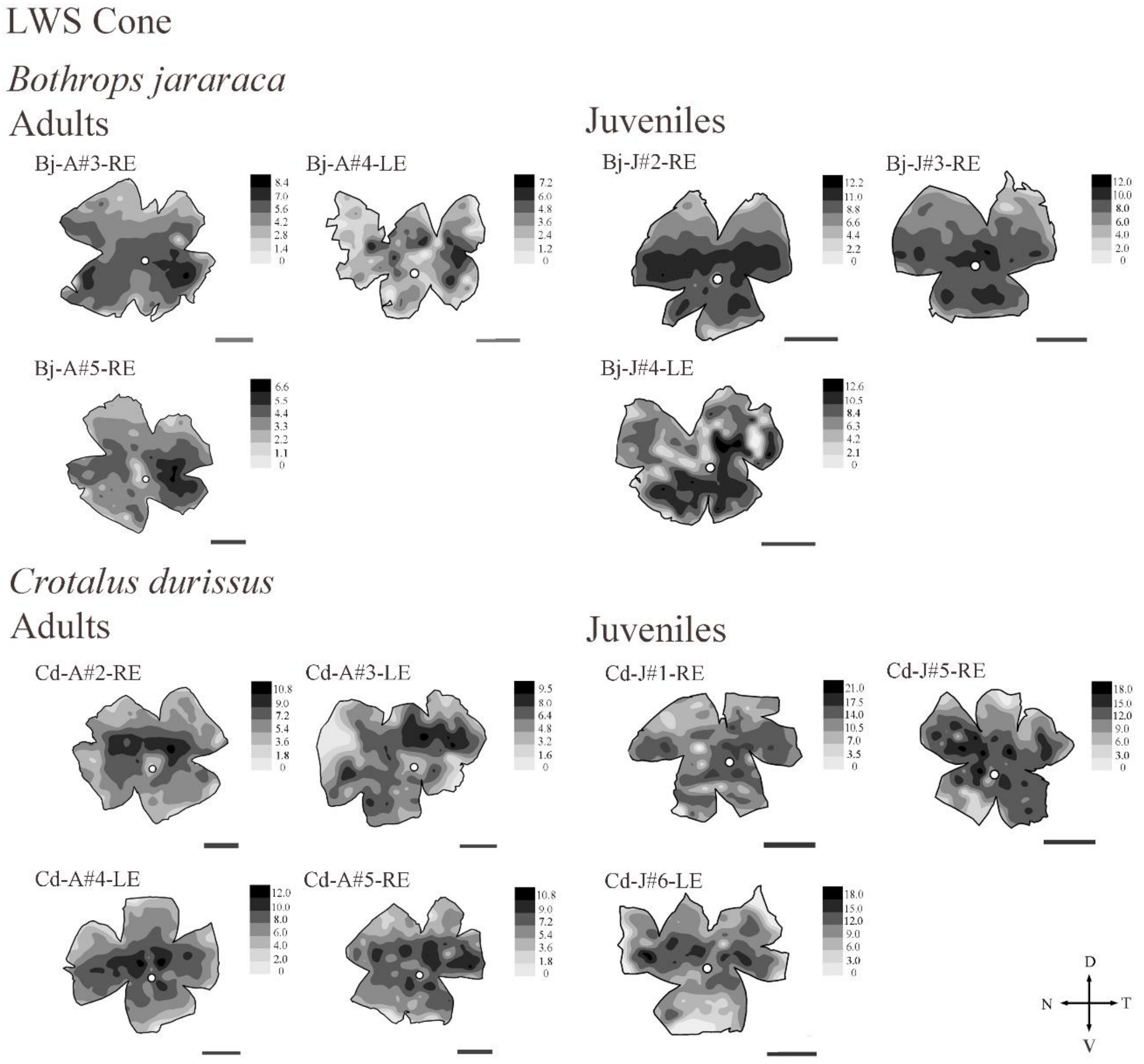

Supplementary Figure 5. Retinal topographic maps of large single and double LWS cones of adults and juveniles of *B. jararaca* and *C. durissus*. In adults of *B. jararaca* the LWS cones form poorly defined visual streaks with the peak density in the ventral-temporal retina. In juveniles of *B. jararaca* the LWS cones are concentrated in a central-ventral retina. In retinas of adults and juveniles of *C. durissus* the LWS cones are arranged in poorly defined visual streaks. Gray bars indicate the density of cells per mm^2, and the values should be multiplied by 10^3. The optic nerve head is depicted as a white circle. D, dorsal; T, temporal; N, Nasal; V, Ventral. Scale bars 2 mm.

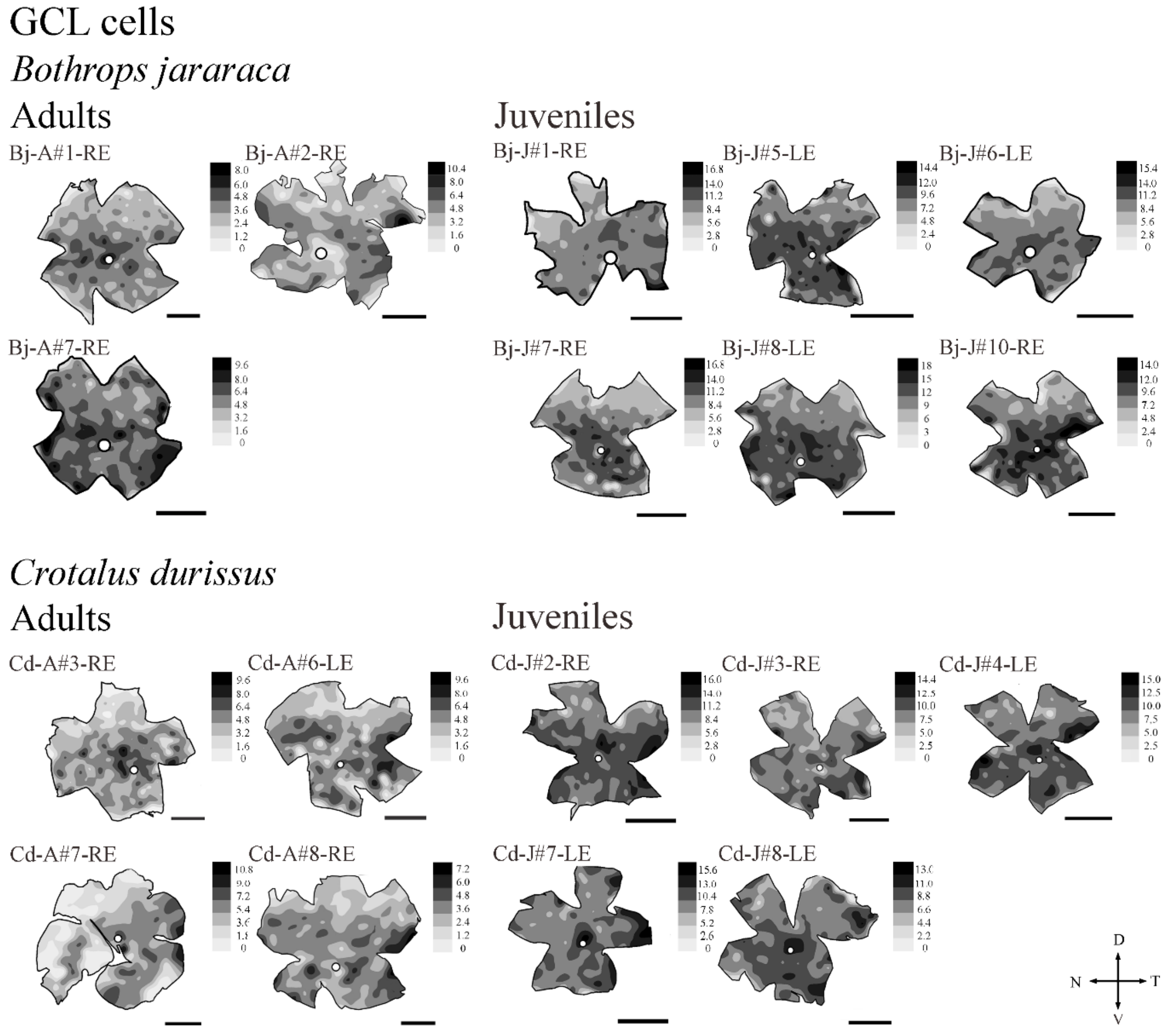

Supplementary Figure 6. Retinal topographic maps of the GCL cells of adults and juveniles of *B. jararaca* and *C. durissus*. In adults of *B. jararaca* and in adults and juveniles of *C. durissus* a diffuse distribution is observed and peak densities are located in the temporal retina. In retinas of juveniles of *B. jararaca* higher densities are located in the ventral retina. Gray bars indicate the density of cells per mm², and the values should be multiplied by 10³. The optic nerve head is depicted as a white circle. D, dorsal; T, temporal; N, Nasal; V, Ventral. Scale bars 2 mm.
